# Exceptional catalytic effects of black phosphorus quantum dots in shuttling-free lithium sulfur batteries

**DOI:** 10.1038/s41467-018-06629-9

**Published:** 2018-10-09

**Authors:** Zheng-Long Xu, Shenghuang Lin, Nicolas Onofrio, Limin Zhou, Fangyi Shi, Wei Lu, Kisuk Kang, Qiang Zhang, Shu Ping Lau

**Affiliations:** 1 Department of Applied Physics, The Hong Kong Polytechnic University, Hong Kong, China; 2 Department of Materials Science and Engineering, Research Institute of Advanced Materials (RIAM), and Center for Nanoparticle Research at Institute of Basic Science (IBS), Seoul National University, Seoul, 08826 Republic of Korea; 3 Department of Mechanical Engineering, The Hong Kong Polytechnic University, Hong Kong, China; 4 Beijing Key Laboratory of Green Chemical Reaction Engineering and Technology, Department of Chemical Engineering, Tsinghua University, 100084 Beijing, China

## Abstract

Lithium sulfur batteries with high energy densities are promising next-generation energy storage systems. However, shuttling and sluggish conversion of polysulfides to solid lithium sulfides limit the full utilization of active materials. Physical/chemical confinement is useful for anchoring polysulfides, but not effective for utilizing the blocked intermediates. Here, we employ black phosphorus quantum dots as electrocatalysts to overcome these issues. Both the experimental and theoretical results reveal that black phosphorus quantum dots effectively adsorb and catalyze polysulfide conversion. The activity is attributed to the numerous catalytically active sites on the edges of the quantum dots. In the presence of a small amount of black phosphorus quantum dots, the porous carbon/sulfur cathodes exhibit rapid reaction kinetics and no shuttling of polysulfides, enabling a low capacity fading rate (0.027% per cycle over 1000 cycles) and high areal capacities. Our findings demonstrate application of a metal-free quantum dot catalyst for high energy rechargeable batteries.

## Abstract (web summary)

Lithium sulfur batteries are promising for next-generation energy storage, but are hindered by polysulfide shuttle effects. Here the authors use black phosphorus quantum dots to adsorb and catalyze the conversion of lithium polysulfides to lithium sulfide, achieving low capacity fade and high sulfur loading.

## Introduction

The development of portable electronic devices drives a search for energy storage systems with lower cost, higher energy density, and better safety than current Li-ion batteries. Among alternative batteries, lithium-sulfur batteries (LSBs) generate interest due to advantages of a high energy density of 2600 W h kg^−1^ and the low cost of sulfur feedstock^1,2^. However, the practical application of LSBs has been plagued by several fundamental challenges^3–5^, including the insulating nature of sulfur and lithium sulfides, the large volume expansion of sulfur, and the diffusion of lithium polysulfides (LiPSs) intermediates during charge/discharge cycles. The last obstacle further induces shuttling effects, self-discharge phenomena, and re-distribution of active particles, leading to rapid capacity degradation. Therefore, achieving the confinement of polysulfides in cathodes is a high priority among the primary research of LSBs.

Research over the past decade has brought progress in stabilizing LiPSs by physical and chemical immobilization approaches. Carbon nanomaterials have been systematically designed with high electrical conductivities, desirable pores, and controlled dimensions to host sulfur particles to physically confine LiPSs^6–9^. Unfortunately, weak interactions between non-polar carbon and polar LiPSs lead to failure in long-term performance of LSBs, causing gradual capacity decay and poor rate performance^10^. Chemical trapping of LiPSs with a polar host^11^, such as N-doped carbon^12^ and metal dichalchogenide^13^, has proven effective for preventing diffusion of LiPSs via polar–polar interactions^14^ or Lewis acid-based bonding.^15^ However, in most cases, chemical immobilization is rather limited for improving battery performance because the blocked LiPSs cannot be effectively reused^16,17^. For example, LiPSs can be strongly adsorbed by TiO_2_, but full conversion to Li_2_S is difficult due to the low electrical conductivity of TiO_2_^18^. Although polar, conductive chemical traps, such as CoS_2_^19^ and WS_2_^20^, have been prepared as sulfur hosts, the high gravimetric density of metal-containing compounds and the limited number of adsorption sites discourages high energy density of sulfur cathodes. Therefore, the effective trapping and conversion of LiPSs remain as important challenges toward achieving high-performance LSBs.

Black phosphorus (BP) is the most thermodynamically stable allotrope of phosphorus, with low resistivity ranging between 0.48 and 0.77 Ω cm^21^ and an exceptionally high room-temperature hole mobility of ~1000 cm^2^ V^−1^ s^−1^ ^22^. BP also possesses a low density of 2.69 g cm^−3^ ^23^, a good bulk conductivity of ~3 S cm^−1^ ^24^, a fast Li-ion diffusion constant^25^, and high binding energies with sulfur^26^. These properties suggest that BP can chemically bind with LiPSs and convert them immediately to Li_2_S through good electrical conductivity and fast Li-ion diffusion, without discernibly compensating the mass fraction of active materials. Few-layered BP flakes have been adopted in separators^26^ or current collectors^27^ to suppress the diffusion of LiPSs, leading to enhanced cyclic stability of LSBs. However, to trap flooded LSPs by the terrace sites of micro-sized BP flakes, a high loading (≈1 wt% with respect to the sulfur weight in electrodes) of BP was employed^27^. Considering the Li_2_S_6_ catholyte with a theoretical capacity of 1299 mA h g^−1^ that was used in that work, the 10 wt% of electrochemically inert BP may reduce the energy density advantage of LSBs. If edge sites of BP are also effective in trapping LiPSs, the amount of BP additives could be significantly decreased by using nanoscale BP particles, for example, BP quantum dots (BPQDs).

In this contribution, we systematically investigate the catalytic properties of the nanostructured BP and propose a metal-free BPQD as a robust catalyst for advanced LSBs. Based on adsorption and electrocatalytic studies, we find that the edge sites of the nanostructured BP are key to the effective immobilization of LiPSs. The edge-selective catalytic property of the BPQDs is further validated using density functional theoretical (DFT) calculations, where the zig-zag (ZZ) terminated BP flakes present stronger binding energies with Li_2_S at the edges than at terrace sites. These results suggest that adsorptivity of LiPS in BP can be largely increased by downsizing BP flakes to QDs. As a proof of concept, we integrate a small amount of BPQDs (2 wt% of the cathode weight) with a sulfur/porous carbon fiber cathode, the LSBs exhibit no diffusion of polysulfides as well as excellent battery performance, including high-rate capability (784 mA h g^−1^ at 4 C) and exceptional cyclic stability (0.027% capacity fade per cycle for 1000 cycles). Finally, an impressive capacity retention of near 90% is observed for high sulfur loading cathodes (up to 8 mg cm^−1^) for 200 deep cycles under lean electrolyte conditions.

## Results

### Rational design of a black phosphorus catalyst

Two-dimensional (2D)-layered crystals usually exhibit strong interatomic covalent bonding and weak interlayer van der Waals interactions, rendering different chemical activities of the atoms at terrace and edge sites. The edge sites tend to possess higher electrochemical catalytic activity than the terrace counterparts in catalysis due to their under-coordinated atomic structures^28–31^. Understanding edge-selective catalytic feature is beneficial in designing catalysts by downsizing the particle size to introduce more active sites. BP is a single-element layered material with one phosphorus atom bonded to three adjacent neighbors to form a honeycomb network, similar to graphite and metal dichalcogenides.

To explore whether BP also possesses such unique edge-selective catalytic properties in LSBs, we rationally prepared different-sized BP flakes through probe sonication and centrifugation of bulk BP crystals in *N*-methyl-2-pyrrolidone (NMP) solution^32,33^. The as-obtained BP products were designated as BP-4K, BP-8K according to their centrifugation rates of 4000 and 8000 rpm, respectively, and BPQD (centrifuged at 12,000 rpm). It is noted that the concentrations of the three BP solutions were controlled as about 0.55 mg mL^−1^, as determined by inductive coupled plasma with atomic emission spectroscopy (ICP-AES)^23^.

**Fig. 1:**
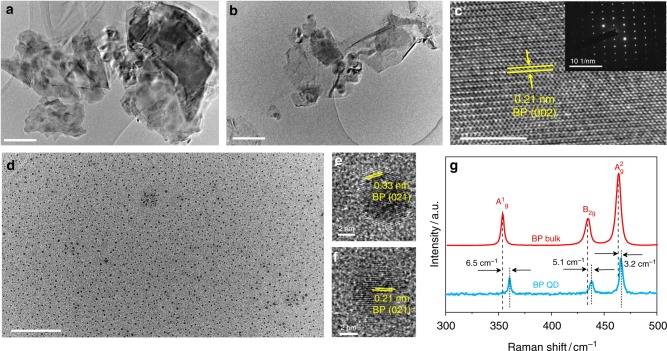
Morphological and structural characterization of the black phosphorus flakes. **a** Transmission electron microscopy (TEM) image of black phosphorus (BP) that was centrifuged at 4000 rpm (BP-4 K), **b** TEM image of BP centrifuged at 8000 rpm (BP-8K), **c** high-resolution TEM (HRTEM) image and corresponding selected area electron diffraction (SAED) pattern (inset) of BP-8K, **d**–**f** TEM and HRTEM images of black phosphorus quantum dots (BPQDs) with monodispersion and high crystallinity, **g** Raman spectra of bulk BP (red) and BPQD (blue) on a silicon (Si) substrate. Scale bars, 200 nm (**a**, **b**); 5 nm (**c**); 10 nm^−1^ inset (**c**); 100 nm (**d**); 2 nm (**e**, **f**)

Figure 1a and b shows the transmission electron microscopy (TEM) images of the BP-4K and BP-8K flakes, which reveal sizes of about 800 and 300 nm, respectively, indicating that smaller flakes were obtained at higher centrifugation rate. The structural features of the BP flakes were investigated by high-resolution TEM (HRTEM) and the selected area electron diffraction patterns (SAED). As shown in Fig. 1c, the lattice constant of 0.21 nm is indexed to (002) plane of BP crystal^34^, consistent with the sharp diffraction spots in the inset. The TEM image of the BPQD in Fig. 1d shows an impressive monodispersion of the BPQDs. The QD size is approximatively 4.5 nm with lattice fringes of 0.33 and 0.21 nm (Fig. 1e, f), assigned to (021) and (002) planes of BP crystal^32,33^, respectively. The dimensions of the three BP samples were also characterized with atomic force microscope (AFM). Supplementary Fig. 1 shows the BP-4K, BP-8K, and BPQD with thickness × particle size of about 6.1 × 800 nm, 4.3 × 300 nm, and 2.5 × 4.5 nm, respectively. Based on the dimensions of different-sized BP flakes, we can roughly estimate the exposed edge density by *S*_edge_/*S*_whole_, where *S*_edge_ and *S*_whole_ refer to the area of exposed edge and the whole surface, respectively. For simplicity, we set the flake as a rectangle prism with two equal edge lengths and QD as a cylinder. The BP-4K, BP-8K and BPQD present *S*_edge_/*S*_whole_ of 1.5%, 2.8%, and 52.6%, respectively, implying the highest exposed edge density of QD structure^35^. The chemical structure of the BPQDs was further probed by Raman spectroscopy. Figure 1g shows three featured Raman peaks at 360.8, 438.1, and 466.2 cm^−1^, referring to A_g_^1^, B_2g_, and A_g_^2^ modes, respectively. Compared to the bulk BP, the three modes of the BPQD are red-shifted by around 6.5, 5.1, and 3.2 cm^−1^, respectively, probably due to the thin thickness and small lateral dimensions of the QDs^23,32,33^.

### Exploration of catalytic effects of black phosphorus flakes

**Fig. 2:**
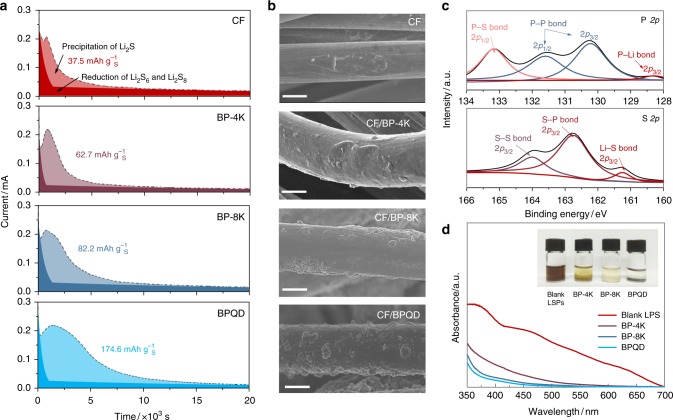
Black phosphorus flakes for lithium polysufide conversion and adsorption. **a** Potentiostatic discharge curves of Li_2_S_8_ tetraglyme solution on different substrates at 2.05 V. The dark/light colors indicate the reduction of Li_2_S_8_/Li_2_S_6_ and the precipitation of Li_2_S, respectively. **b** scanning electron microscopy (SEM) images showing the precipitation of Li_2_S on different substrates as indicated in **a**, **c** X-ray photoelectron spectroscopy (XPS) spectra for P 2*p* and S 2*p* of the black phosphorus (BP) flakes adsorbed with lithium polysulfides (LiPSs), **d** ultraviolet–visible (UV–vis) spectra of LiPS with variation in color upon adsorption by different-sized BP flakes. Scale bar, 5 µm (**b**). (CF is carbon fiber, BP-4K is BP that was centrifuged at 4000 rpm, BP-8K was centrifuged at 8000 rpm, BPQD is black phosphorus quantum dot)

The catalytic effects of BP flakes on the polysulfide redox reaction were investigated by potentiostatic discharge deposition of Li_2_S from Li_2_S_8_ tetraglyme solution on different substrates, including carbon fiber (CF)^35^, CF/BP-4K, CF/BP-8K, and CF/BPQD. The amounts of the BP flakes dispersed on CF substrates were controlled to be the same. Galvanostatic discharge was conducted to 2.06 V to consume most long-chain polysulfides before applying a 0.01 V overpotential to drive the formation of Li_2_S^18,36–38^. Figure 2a exhibits the potentiostatic discharge curves of the aforementioned electrodes at 2.05 V. Fits of the capacity contributions of the polysulfide reduction and the deposition of Li_2_S are represented by dark and light colors, respectively (See detailed information in Supplementary Fig. 2). The capacities of the Li_2_S precipitation on CF, CF/BP-4K, CF/BP-8K, and CF/BPQD are determined to be 37.5, 62.7, 82.2, and 174.6 mA h g^−1^, respectively, based on the sulfur weight in catholyte. It manifests that the Li_2_S deposition capacity increases when decreasing BP flake size.

The morphology of the precipitated Li_2_S on different substrates was observed by scanning electron microscopy (SEM). As shown in Fig. 2b, the Li_2_S on CF surface is a discrete coating, suggesting insufficient deposition of the Li_2_S due to the poor affinity between polysulfides and non-polar carbon. This deposition type is ascribed to a very high energy barrier for the redox reaction of polysulfides on CF^36,38^. The amounts of the Li_2_S deposited on the CF/BP-4K and CF/BP-8K are significantly improved with a uniform deposition. The enhanced precipitation of the Li_2_S can be attributed to the strong interactions between polysulfides and the conductive polar BP flakes^26,27^, thus lowering the precipitation energy barriers and increasing nucleation sites. Interestingly, we observe radial growth of three-dimensional (3D) Li_2_S particles on the CF/BPQD, in agreement with the highest Li_2_S precipitation capacity depicted in Fig. 2a. In general, the precipitation of the Li_2_S on carbon substrate follows a 2D lateral growth theory along the polysulfide-Li_2_S-substrate tri-phase boundary^37^. The 3D growth of Li_2_S was only observed in LSBs with TiC catalyst^36^ and an organic redox mediator^38^, which promoted nucleation/growth of Li_2_S along the radial direction by regulating the impingement of insulating Li_2_S. Analogically, the BPQDs facilitate 3D precipitation of the Li_2_S by the strong adsorption and rapid conversion of LiPSs on the numerous catalytically active sites. Compared to the routine 2D deposition of Li_2_S, such anomalous 3D structure of Li_2_S enables higher usage of LiPSs in cathodes, thus more effectively suppressing the diffusion of LiPSs to bulk electrolyte. The above results suggest the effective catalytic effect of the BP flakes in LiPS conversion reaction and that BPQDs outperform the larger BP flakes.

In addition, the catalytic effect of BPQDs was further verified via linear sweep voltammetry (LSV) and symmetric cyclic voltammetry (CV) measurements of the CF and CF/BPQD electrodes in the presence of Li_2_S_4_ catholyte (Supplementary Fig. 3)^30,39,40^. The CF/BPQD electrodes exhibited higher reaction peaks in LSV curves and a larger exchange current density of 98 µA cm^−2^ than 75 µA cm^−2^ for CF calculated from Tafel plots. These results further evidence that the kinetics of polysulfide redox reactions are effectively improved by BPQDs.

To disclose the reason why BPQDs exhibited superior catalytic property, we examined the interactions between LiPSs and BP flakes through X-ray photoelectron spectroscopy (XPS) and ultraviolet–visible (UV–Vis) absorption measurements. The nature of the interactions between LiPSs and BPQDs was probed by XPS as shown in Fig. 2c. In the deconvoluted P 2*p* spectra, apart from the two P–P bonds at 130.1 eV and 131.6 eV referring to P 2*p*_3/2_ and P 2*p*_2/1_ signals in pristine BPQD (Supplementary Fig. 4)^26^, two additional peaks at 128.3 eV and 133.0 eV, corresponding to P-Li 2*p*_3/2_ and P-S 2*p*_2/1_, are detected. Moreover, a strong S-P 2*p*_3/2_ bond at 162.7 eV is observed in S 2*p* spectrum. These results indicate that the BPQDs strongly interact with LiPSs via both P–S and P-Li bonds, resulting in high adsorptivity between the materials. Static adsorption of the LiPSs was conducted by adding same amounts (about 1 mg) of BP flakes separately in 10 mL Li_2_S_8_ tetraglyme solution, which showed distinct bulk adsorptivity with a sequence of BPQD > BP-8K > BP-4K (inset Fig. 2d), consistent with the UV–Vis spectra results. The different LiPS adsorptivities are related to the population of the active sites, suggesting the most adsorbing sites on the BPQDs among the three BP samples. Considering that the difference of terrace areas for the BP flakes with the same mass is relatively trivial, the dramatically increased number of edge sites on the QDs are suggested to contribute to the significant difference in adsorptivity.

**Fig. 3:**
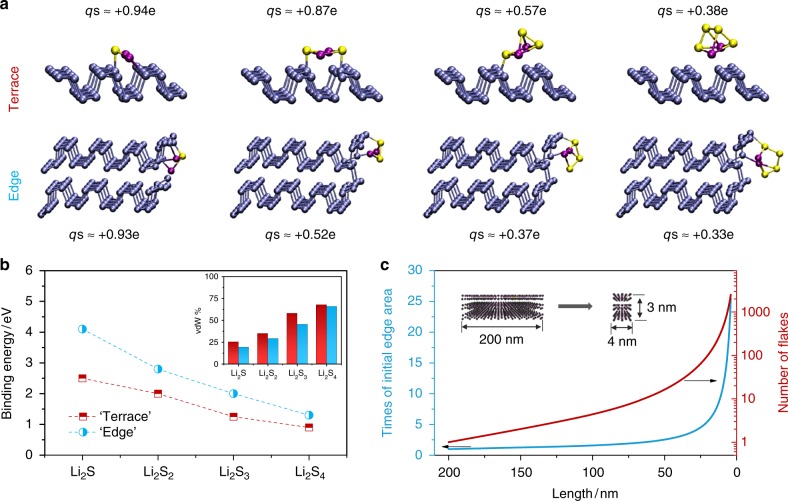
Calculation of lithium polysulfide adsorption on black phosphorus flakes. **a** Snapshots of lithium polysulfides (LiPSs) adsorbed on terrace and edge sites of black phosphorus (BP) monolayer and nanoribbons, respectively. The partial charge on the sulfur atom is defined as *qs*, **b** binding energies of LiPSs adsorbed on terrace and edge sites of BP, the inset shows the fraction of van der Waals (vdW) contribution to the bond, **c** the increase of exposed edge area and the number of flakes by downsizing a large BP flake to black phosphorus quantum dots (BPQDs)

To quantify the relative adsorptivity between terrace and edge sites of the BPQDs with LiPS, we performed DFT calculations. Figure 3a shows snapshots of the atomic structures of Li_2_S_*n*_ (*n* = 1–4) adsorbed on the terrace sites of free-standing BP monolayer as well as adsorbed on edge sites of bilayers BP nanoribbons. Figure 3b presents the binding energies, giving rise to two interesting results: (i) the binding energy of Li_2_S_*n*_-BP decreases when the size of the adsorbed molecule increases^26,27^; (ii) the binding energies of Li_2_S_*n*_ adsorbed at the edge of BP nanoribbons are significantly larger than those at terrace sites. Binding energies computed with and without van der Waals (vdW) correction indicate that the vdW contribution to bonding (i.e., the binding energy without vdW is divided by the binding energy with vdW) is always smaller when the LiPS is adsorbed at edge sites (see inset of Fig. 3b). Bader charge analysis^41^ shows that in average, partial charges on Li atoms are similar whether the LiPS is adsorbed at terrace or edge sites, equaling to −0.86 eV. However, we found lower partial charges on S-atom part of LiPS adsorbed at edge sites, suggesting less ionic character of the bond. The stronger covalent character of bonds formed between BP edges and LiPS can be attributed to the high reactivity of the under-coordinated P-atoms at the zig-zag terminated edges compared to stoichiometric P-atoms at the terrace^42,43^. In addition, the exposed edge area of BP flakes can be rationally increased by downsizing BP. For example, when a BP rectangle prism with two equal edge lengths of 200 nm and a thickness of 3 nm is divided into BPQDs with the same thickness and equal edge lengths of 4 nm, the exposed edge surface area increases by ~25 times (Fig. 3c), in agreement with the improved *S*_edge_/*S*_whole_ ratio from BP-8K to BPQD. At the same time, the number of particles increases from 1 to 2500, with largely decreased particle size, ensuring uniform dispersion of catalysts within host materials. Together with the above experimental results, we can conclude that BP flakes exhibit an edge-selective catalytic property. Further, a QD structure with a high aspect ratio and numerous edge sites should be an ideal choice to improve LiPS adsorptivity and redox reaction kinetics in LSBs.

### Integrate black phosphorus quantum dots with sulfur/carbon cathodes

**Fig. 4:**
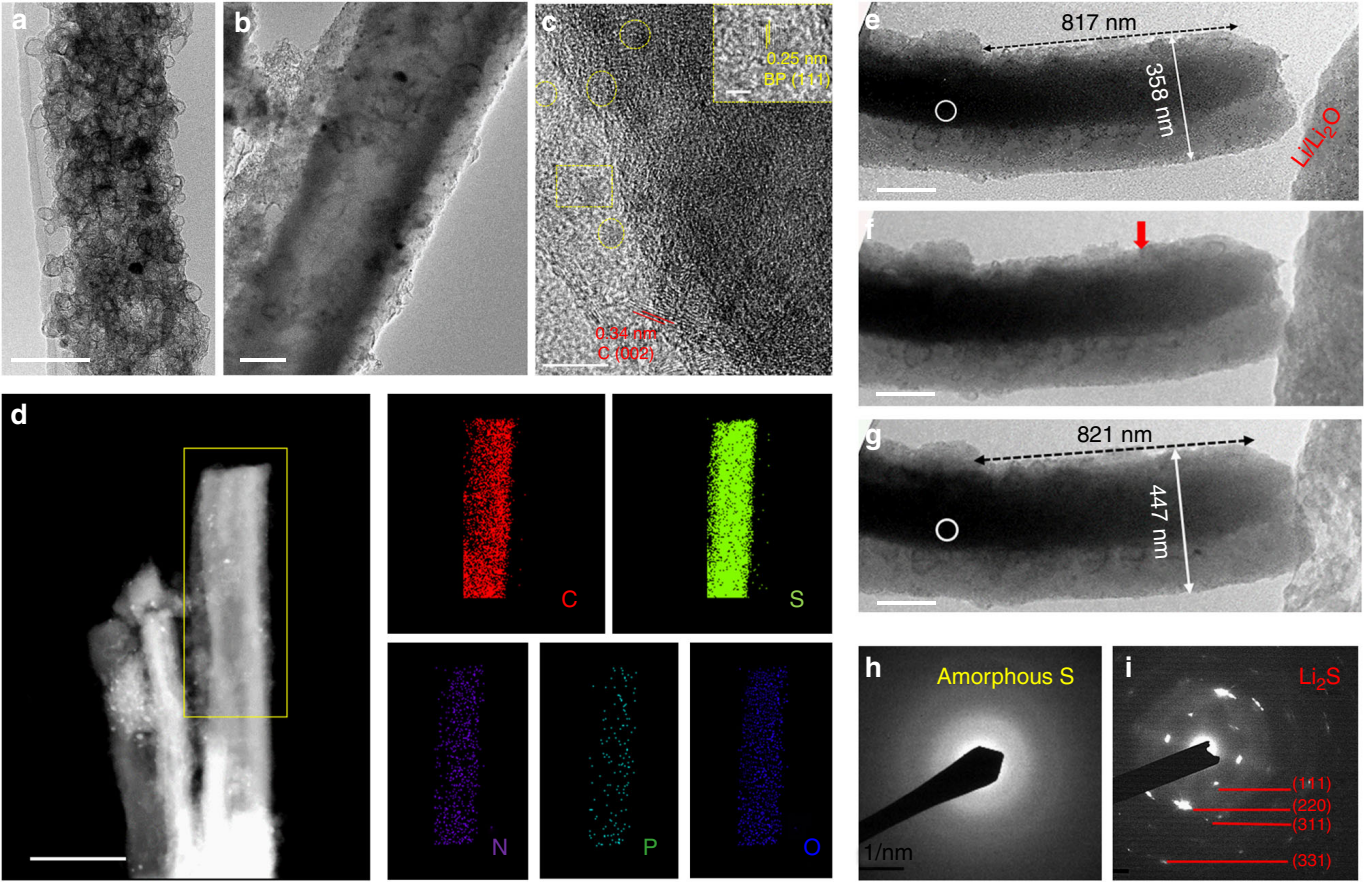
Integrate black phosphorus quantum dots with porous carbon/sulfur cathodes. **a** Transmission electron microscopy (TEM) image of the porous carbon nanofiber (PCNF) consisting of numerous hollow carbon spheres, **b** TEM image of the porous carbon host in **a** filled with sulfur particles and black phosphorus quantum dot (BPQD), designated as PCNF/S/BPQD, **c** high-resolution TEM (HRTEM) image showing the graphitic carbon layers of the PCNF and the uniform dispersion of BPQD within PCNF/S/BPQD, **d** scanning tunneling electron microscopy (STEM) image and energy dispersive spectroscopy (EDS) elemental mapping of the PCNF/S/BPQD fibers, **e**–**g** TEM images captured during lithiation of a PCNF/S/BPQD fiber, the red arrow in **f** refers to the reaction front, **h**, **i** selected area electron diffraction (SAED) patterns of the selected area with white circles in **e** and **g**, respectively. Scale bars, 100 nm (**a**); 200 nm (**b**); 10 nm (**c**); 2 nm inset (**c**); 500 nm (**d**); 200 nm (**e**–**g**)

To apply the BPQD catalyst in actual working cathodes, we integrated the BPQDs and sulfur particles with porous carbon nanofibers (PCNFs). The PCNFs are consist of numerous hollow graphitic carbon spheres with open pores (See Methods and Supplementary Fig. 5), allowing the impregnation of sulfur particles and BPQDs inside.^44^ It is noted that the PCNF host possesses a high pore volume of 0.82 cm^3^ g^−1^ and a large surface area of 1277 m^2^ g^−1^, both of which are important in delivering high sulfur loading. Figure 4a and b shows TEM images of the PCNF and PCNF/S/BPQD, respectively. Obviously, the hollow carbon spheres in the PCNF are filled with sulfur particles. The HRTEM image of the PCNF/S/BPQD in Fig. 4c presents uniformly dispersed BPQD crystals with a fringe space of 0.25 nm as well as graphitic carbon layers with a lattice range of 0.34 nm. The scanning TEM (STEM) image and energy dispersive spectroscopy (EDS) elemental mapping of an individual fiber presented in Fig. 4d shows that (i) sulfur particles are uniformly integrated with the PCNF host and (ii) BPQDs are homogeneously dispersed among sulfur particles. It is noted that nitrogen doping is also observed in the PCNF/S/BPQD composite, which is derived from the polyacrylonitrile polymer precursor^45,46^. The chemical components of the ternary hybrid material were determined by thermal gravimetric analysis (TGA) to be about 68 wt% for sulfur and 2 wt% for BPQDs as shown in Supplementary Fig. 6. PCNF/S composites were also prepared for control experiments.

### Electrochemical behaviors of black phosphorus quantum dots/sulfur/carbon cathodes

The volume expansion of sulfur particles after being fully lithiated to Li_2_S is theoretically calculated to be 80%. This large volume variation would squeeze lithiated product out of porous carbon host^47^, aggravating the polysulfide diffusion and the capacity degradation of sulfur cathodes. To identify whether the BPQDs can suppress polysulfide diffusion even under large volume expansion of sulfur we conducted a series of in situ experiments, including TEM, transmission battery (TB) and electrochemical impedance spectroscopy (EIS) measurements. In situ TEM investigation was performed by applying an external voltage of −2 V to the PCNF/S/BPQD working electrode versus Li/Li_2_O counter electrode. The gradual structural evolution due to the lithiation of the PCNF/S/BPQD fiber is recorded in Supplementary Movie 1, from which the captured images at different reaction stages are shown in Fig. 4e–g. It is worth noting that the lithiation process is uniform over the circular cross-section from the contact interface to the PCNF/S/BPQD fiber direction. The SAED patterns indicate that amorphous sulfur particles (Fig. 4h) are fully converted to crystalline Li_2_S (Fig. 4i) after lithiation^48,49^. To measure the volume expansion, an 817 nm long fiber with a diameter of 358 nm was selected, which expanded to 821 nm in length and 447 nm in diameter after full lithiation, corresponding to 56.7% volume expansion. Although this value is lower than the theoretical 80%, HRTEM image shows that the lithiated product spilled over the carbon spheres due to the volume expansion (Supplementary Fig. 7). In real batteries, a 56.7% volume expansion would induce overflowing of polysulfides and shuttling effect. As a direct evidence, the in situ TB experiments show that the colorless electrolyte around the PCNF/S changed to yellow due to overflowing of polysulfides during discharging (Supplementary Fig. 8), in sharp contrast to the unchanged color of electrolyte in the PCNF/S/BPQD battery, suggesting effective immobilization of LiPSs by BPQDs.

In situ EIS is a powerful technique to probe the electrochemical impedance of LSBs at different reaction stages^50,51^. Nyquist plots of the PCNF/S/BPQD and the PCNF/S cathodes were collected during the initial lithiation process. The system resistance (*R*_s_), electrolyte/electrode interface resistance (*R*_suf_), and charge transfer resistance (*R*_ct_) obtained from equivalent circuit are plotted in Supplementary Fig. 9. It is noteworthy that *R*_ct_ of the PCNF/S is greatly increased as compared to that of the PCNF/S/BPQD, implying diffusion of the LiPSs and shuttling effect of the PCNF/S cathode. For the PCNF/S/BPQD electrode, both *R*_suf_ and *R*_ct_ remained stable throughout the whole discharge process, indicating that the BPQDs can suppress the outward diffusion of the LiPSs and maintain structural integrity. These findings offer solid evidences for the effective immobilization of the LiPSs by the BPQDs in porous carbon/sulfur electrodes.

**Fig. 5:**
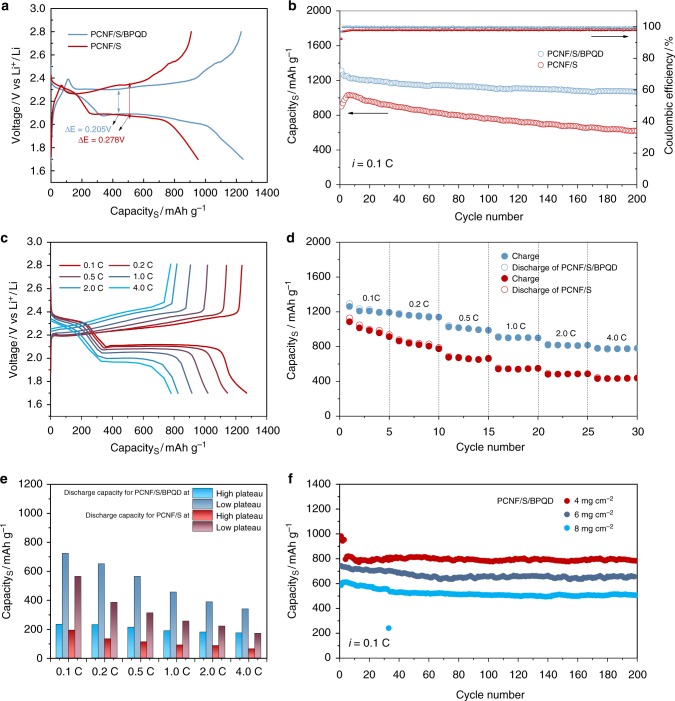
Electrochemical performance of cathodes. **a** Initial discharge–charge profiles at 0.1 C between 1.7 and 2.8 V vs. Li^+^/Li, **b** cyclic capacities and Coulombic efficiencies at 0.1 C for 200 cycles, **c** discharge–charge voltage profiles of the porous carbon nanofiber/sulfur/black phosphorus quantum dot (PCNF/S/BPQD) cathode at different rates, **d** cyclic capacities at different rates, **e** high plateau and low plateau discharge capacities derived from rate performance, **f** cyclic performance of the PCNF/S/BPQD at 0.1 C for 200 cycles with increasing sulfur loadings of 4, 6, and 8 mg cm^−2^

The electrochemical performance of the PCNF/S/BPQD electrodes were evaluated between 1.7 and 2.8 V vs. Li^+^/Li using lithium metal as counter electrode; for comparison, the PCNF/S electrodes were also tested. It is noted that the lithiation of the BP occurs below 1.2 V vs. Li^+^/Li^52^, thus the BPQDs will not participate in any reaction in the 1.7–2.8 V voltage range. Figure 5a presents the initial discharge/charge profiles of the PCNF/S and PCNF/S/BPQD electrodes at 0.1 C (1 C = 1675 mA g^−1^). The two discharge plateaus at ~2.4 and 2.1 V are associated with the reduction of sulfur particles to long-chain polysulfides and the subsequent conversion of soluble polysulfides to insoluble lithium sulfides, respectively^44–46^. The reduction of the lithium sulfides to sulfur is presented by the plateau at 2.3–2.4 V in the corresponding charge curve. Figure 5a evidently shows that (i) the reversible specific capacity from the PCNF/S/BPQD electrode is much larger than that of the PCNF/S (1234 vs. 907 mA h g^−1^, respectively); (ii) the PCNF/S/BPQD electrode presents a much lower polarization between discharge and charge curves than that for the PCNF/S (0.205 vs. 0.278 V, respectively). The higher specific capacity of the PCNF/S/BPQD implies that the sulfur utilization is significantly enhanced by the BPQD via trapping and reusing polysulfides^18,27,39^. The lower polarization suggests higher redox reaction kinetics in the LSB, consistent with the higher exchange current densities mentioned before. In addition, the CV curves of the PCNF/S/BPQD electrodes (Supplementary Fig. 10) show that the low-voltage reduction peak (referring to Li_2_S_4_ → Li_2_S) shifts to higher potential and the oxidation peak shifts to lower potential than the PCNF/S, suggesting the catalytic effect of the BPQD on enhancing the oxidation/reduction kinetics of sulfur particles^18^.

Figure 5b shows the cyclic stability of the PCNF/S/BPQD and PCNF/S electrodes at a low current density of 0.1 C. The PCNF/S/BPQD exhibited an initial capacity of 1234 mA h g^−1^ and maintained at 1072 mA h g^−1^ after 200 cycles, rendering an ultralow capacity fading rate of 0.06% per cycle. In contrast, the PCNF/S delivered a climax capacity of 1036 mA h g^−1^ and maintained at 623 mA h g^−1^ after 200 cycles, leading to a high-capacity fading rate of 2% per cycle. The rapid capacity degradation of the PCNF/S can be evidenced by its lower Coulombic efficiencies during cycles (≈98% for PCNF/S vs. ≈99.9% for PCNF/S/BPQD, respectively). The two electrodes were also cycled in LiNO_3_-free electrolyte (Supplementary Fig. 11). Although the cyclic capacities for PCNF/S/BPQD are slightly lower than in electrolyte with LiNO_3_ additive, due to the positive effect of LiNO_3_ in stabilizing Li metal and suppressing the shuttle effect of LPSs^53,54^, the cyclic performance is still much better than PCNF/S electrodes. Considering the similar sulfur loading and the same carbon host for two electrodes, it is evident that the differences in cyclic capacities are attributed to the different redox reaction kinetics and entrapment of LiPSs by the BPQDs, enabling high Coulombic efficiencies and high cyclic capacities. To verify the long-term stability of the PCNF/S/BPQD, the batteries were tested at a low current density of 0.5 C for 500 cycles and a high current density of 2 C for 1000 cycles (Supplementary Fig. 12). It is observed that the PCNF/S/BPQD electrodes present a capacity retention of 74% over 500 cycles at 0.5 C, rendering a capacity fading rate of 0.052%, which is much lower than the 0.163% for PCNF/S. When cycling at 2 C, PCNF/S/BPQD presents the excellent capacities of 810 mA h g^−1^ at 1st cycle and 589 mA h g^−1^ at 1000th cycle, giving rise to an extremely low capacity decay rate of 0.027% per cycle.

The catalytic effect of the BPQDs can be more clearly evaluated by cycling batteries at high rates, which require faster charge transfer and higher redox reaction kinetics at the electrode/electrolyte interface. The PCNF/S/BPQD exhibits reversible capacities of 1266, 1172, 1030, 910, 821, and 784 mA h g^−1^ at 0.1, 0.2, 0.5, 1, 2, and 4 C, respectively (Fig. 5c). Both the discharge and charge curves overlap well below the current density of 1 C, suggesting excellent kinetics and small polarizations of the PCNF/S/BPQD electrodes. The apparent platforms are still maintained even at high rates above 1 C, which can be attributed to the firm chemical interactions between LiPSs and BPQD and the facilitated conversion reactions. The decreased capacity and increased polarization for the PCNF/S/BPQD may be ascribed to the high ohmic resistance at higher current densities^55,56^. In contrast, the PCNF/S displays much lower capacities (Fig. 5d) under the same rate cycling conditions. To elucidate reasons for the difference, we separated out the high plateau (at about 2.35 V, referring to generation of polysulfides) and the low plateau (at about 2.1 V, referring to nucleation/growth of lithium sulfides) discharge contributions to the total capacities at different current rates for the PCNF/S/BPQD and PCNF/S electrodes (Fig. 5c and Supplementary Fig. 10). The high plateau capacity for PCNF/S/BPQD decreases by ca. 25% (from 232 to 173 mA h g^−1^) with current density increasing from 0.1 to 4 C (Fig. 5e), which is much lower than ca. 67 % (from 191 to 63 mA h g^−1^) for PCNF/S, confirming the strong immobilization of LiPSs by BPQD in the PCNF/S/BPQD electrode. We also find that the low plateau discharge capacity at 4 C for the PCNF/S/BPQD maintains 47% of the capacity at 0.1 C, greater than the 30% for the PCNF/S electrodes. The high discharge capacity retention at low plateau indicates the facilitated conversion reactions catalyzed by the BPQD in PCNF/S/BPQD electrodes. In addition, Supplementary Fig. 10 shows the high and low discharge plateau voltages of PCNF/S and PCNF/S/BPQD electrodes at different C-rates. In contrast to the sharp decrease of low discharge plateau voltage for PCNF/S at high rate (i.e., from 2.12 V at 0.1 C to 1.76 V at 4 C), the PCNF/S/BPQD system displays much better performance (i.e., from 2.11 V at 0.1 C to 1.99 V at 4 C), indicating the low polarization and fast redox reaction from the catalytic effect of BPQD in LSBs^27^. As a final piece of evidence, the PCNF/S/BPQD electrodes exhibit cyclic capacity retentions of 95%, 90%, and 89% after 200 cycles at 0.1 C at high areal sulfur loadings of 4, 6, and 8 mg cm^−2^ (Fig. 5f), respectively. The impressive stability at high sulfur loadings suggests that the BPQDs can effectively immobilize flooded polysulfides of thick electrodes. It is noteworthy that the 89% capacity retention for PCNF/S/BPQD cycled under an electrolyte/sulfur (E/S) ratio of 6.5 mL g^−1^ for 200 cycles is competitive to the state-of-the-art LSBs with low E/S ratios^10,57^. It is believed that better performance under lean electrolyte conditions can be achieved by combining our findings of using BPQD catalysts and optimizations in other components of LSBs^58,59^. Overall, the excellent electrochemical performance of the PCNF/S/BPQD demonstrates that the BPQDs are highly effective in improving LSB performance with sulfur/carbon cathodes.

## Discussion

We have demonstrated the catalytic effect of the BPQDs for efficient trapping and conversion of LiPSs via a suite of experimental and theoretical studies. To identify the superiority of BPQDs to other reported catalysts for LSBs, we have compared the physicochemical properties and their corresponding electrochemical performance in LSBs in Supplementary Table 1 and Table 2. The unique advantages of the BPQD catalyst for LSBs can be summarized as following: (i) the BPQD as a metal-free catalyst holds the lowest gravimetric density among reported catalysts (Supplementary Table 1) to the best of our knowledge, thus for the same weight fraction of catalyst, the active surface area will far exceed that of metals (Pt, Ni)^39,40^, metal oxide phases (Fe_2_O_3_, MnO_2_)^60,61^ and metal nitride materials (VN, TiN)^56,62^. (ii) The BPQDs with unique edge-preferential LiPS immobilization ability offer more active sites than routine 2D structures. The QD structure with exposed edge sites outperforms bulk flakes based on the amount of active sites, thus leading to better electrochemical performance. For example, both WS_2_ and MoS_2_ flakes exhibited an edge-selective catalytic property for LiPSs; however, the reported rate capacities (~400 mA h g^−1^ at 2 C for MoS_2_/CNF/Li_2_S_8_ and 380 mA h g^−1^ at 1 C for WS_2_/Li_2_S_6_)^30^ are much lower than the current BPQD modified cathode (784 mA h g^−1^ at 4 C). (iii) The BPQD with few-layer thickness is expected to possess high electrical conductivity^27^, which is higher than those for metal oxides^60,61^ and metal carbides^36^, thus ensuring rapid conversion kinetics for trapped LiPS to Li_2_S particles. In addition, the physical confinement from the PCNF host also contributes to the excellent electrochemical performance. Post-cycled analysis reveals that the overall morphology of the PCNF/S/BPQD composite was almost intact (Supplementary Fig. 13), indicating the robust structural stability of PCNF host. HRTEM images and elemental mappings show that BPQD crystals and sulfur particles are uniformly confined within the fibers, suggesting the chemical and structural integrity of PCNF/S/BPQD during cycling. Consequently, our PCNF/S/BPQD cathodes exhibit one of the best electrochemical performances among reported catalyst-modified cathodes in terms of cyclic capacities, high-rate capability, and areal capacities at high sulfur loadings (Supplementary Table 2). PCNF/S/BPQD electrodes with a high sulfur loading of 8 mg cm^−2^ and a low E/S ratio of ~6.5 mL g^−1^ delivered an area capacity of 4.4 mA h cm^−2^ after 200 cycles, which is higher than the 4.0 mA h cm^−2^ for commercial LiCoO_2_ cathodes^57^. It is worth noting that high sulfur loading and low E/S ratio are recently considered critical challenges for fabricating practical LSBs to outperform commercial LIBs^10^. We believe that incorporating our highly effective BPQD catalyst with highly concentrated polysulfide catholyte would be a promising strategy to mitigate these issues (Supplementary Fig. 14). Moreover, thanks to the high yield of the top-down synthetic procedure^27^ as well as the low content (2 wt%) needed to boost the LSB performance, the large scale application of BPQDs in LSBs is not problematic.

In summary, we demonstrated the effectiveness of the BPQDs as a catalyst for polysulfide immobilization and conversion in LSBs. By downsizing the particle size of 2D BP flakes, we found that the BPQDs exhibited higher LiPS adsorptivity and larger Li_2_S precipitation capacity than the large BP flakes. DFT calculations revealed that the edge sites of the 2D BP materials showed preferential adsorption of polysulfides, inducing more catalytically active sites than bulk BP flakes, thus dictating the superior catalytic performance of the BPQD. To apply the BPQD in actual cathodes, a small amount of the BPQD was integrated with porous carbon/sulfur composite. Time-sequence TEM images showed a large volume expansion and overflowing of reaction products of the PCNF/S/BPQD fiber during lithiation, however, there was neither diffusion of yellow LiPS in a transparent battery nor an increase in battery impedance for the PCNF/S/BPQD electrodes in EIS measurements, thus demonstrating the effectiveness of the BPQD in immobilizing LiPSs. As a result, the PCNF/S/BPQD electrode exhibited impressive electrochemical performance, including a reversible capacity of 1072 mA h g^−1^ after 200 deep discharge/charge cycles at 0.1 C, a high-rate capacity of 784 mA h g^−1^ at 4 C and remarkable capacity retention of near 90% at high sulfur loadings up to 8 mg cm^−2^. We believe that these findings open a new avenue toward the design of high energy rechargeable batteries through the exploration of metal-free catalyst materials.

## Methods

### Materials preparation

For BPQD preparation, BP crystal was purchased from Smart Elements. We added 45 mg BP crystals into 20 mL NMP solution and ultra-sonicated using an ultrasonic bath (400 W) at a temperature of 20 °C through the whole experiment for 8.0 h. The obtained BP solution was purified by centrifugation, with rates of 1000–3000 rpm for 30 min, to remove large particles at bottom. Then the BP suspension was further sonicated using probe sonication. The as-obtained BP/NMP solution was centrifuged at 4000, 8000, and 12,000 rpm for 30 min, and the precipitates were collected and designated as BP-4K, BP-8K, and BPQD, respectively. The yield of BPQD produced in this work is discussed in Supplementary Note 1. The as-obtained BP NMP solutions were stored in an Ar-filled glove box to ensure their stability. Without mention, all below experiments related to BP flakes were conducted in the glove box.

For porous carbon nanofiber synthesis, 0.5 g polyacrylonitrile was dissolved in 20 ml *N*,*N*-dimethylformamide solvent and magnetically stirred for 8 h at 80 °C. Then, 1.0 g iron (III) acethylacetonate was added to the above solution and stirred for another 8 h. The polymer mixture was electrospun into nanofibers using an electrospiner at 18 kV with a constant flow rate of 1 mL h^−1^. The neat fibers were stabilized in air for 3 h at 220 °C and sequentially carbonized in Ar atmosphere for 1 h at 650 °C at a ramp rate of 3 °C min^−1^. The resultant carbon nanofibers containing Fe_3_C particles were soaked in fuming HNO_3_ for 10 h to create hollow graphitic carbon spheres via removing the Fe_3_C catalyst. To open the graphitic carbon walls, the as-obtained porous carbon nanofibers were mixed with KOH at a mass ratio of 1:4 and transferred to a tube furnace and heated at 750 °C for 0.5 h under Ar gas flow. Then the resulting product was washed with flooded amount of HCl (0.1 M) and DI water subsequently, before drying in vacuum oven for 8 h at 100 °C.

For PCNF/S composite preparation, the PCNF was mixed with sulfur particles at a mass ratio of 25:75. The PCNF/S mixture was then placed in a tube furnace with Ar flow and heated at 155 °C for 12 h to infiltrate molten sulfur within PCNF host. The PCNF/S/BPQD composites were prepared by adding about 3 wt% BPQD in PCNF/S composite in CS_2_ solution. CS_2_ can dissolve sulfur particles thus the BPQD and sulfur would be simultaneously re-infiltrated into PCNF host by capillary force. It is worth noting that the morphology and stability of BPQD would not be affected by CS_2_ solvent (Supplementary Fig. 15).

### Materials characterization

For structural characterization of BP, Raman spectra were obtained from a Horiba Jobin Yvon HR800 Raman microscopic system equipped with a 488 nm laser operating at 180 mW. The spot size of Raman laser was controlled near 1 µm. A TEM (JEOL 2100F) working at 200 kV was used to estimate the morphological information of BP flakes and BPQD.

For PCNF and PCNF/S/BPQD characterization, the morphologies were investigated using a SEM (JEOL 6700) and a TEM. The STEM image and elemental mapping of PCNF/S/BPQD were conducted on the JEOL 2100-TEM equipped with EDS detector. The surface area and pore size distribution of PCNF host were determined from N_2_ adsorption/desorption isotherms at 77 K using an automated adsorption apparatus (Micromeritics, ASAP 2020). The chemical compositions of sulfur/carbon composites were evaluated by TGA (Q5000) at a ramp rate of 5.0 °C min^−1^ in nitrogen atmosphere.

### Adsorption and catalytic studies of black phosphorus quantum dots

Li_2_S_8_ solution was prepared by mixing sulfur particles and Li_2_S with a molar ratio of 7:1 in tetraglyme solvent, followed by stirring at 50 °C for 8 h in an Ar-filled glove box. 1 mg of BP-4K, BP-8K, and PBQD powders were dispersed individually in 10 mL Li_2_S_8_/tetraglyme solution with a concentration of 5 mmol L^−1^ in sulfur. To observe the color change, the mixtures were kept standing for 12 h. The supernatant and the BP precipitates of the mixtures were studied by UV–vis spectrophotometry and XPS, respectively. The precipitates were obtained by centrifugation.

The nucleation and growth of Li_2_S from soluble polysulfides were studied by potentiostatic deposition of Li_2_S_8_ tetraglyme solution (0.2 mol L^−1^ based on sulfur) on CF-based current collectors. CF papers were punched into disks with a diameter of 14 mm and about 0.40 mg of BP-4K, BP-8K, and BPQD powders were separately dispersed on CF papers using pure ethanol as solvent. 25 µL Li_2_S_8_ was dropped onto the CF/BP current collectors as cathode. Lithium foil was employed at the counter electrode, which was separated with cathode by Celgard 2400 membrane and dropped with 25 µL 0.50 mol L^−1^ lithium bis(trifluoromethanesulfonyl)imide (LiTFSI) tetraglyme electrolyte on the Li metal side. The cells were galvanostatically discharged to 2.06 V at a constant current density of 0.112 mA, and then kept potentiostatically at 2.05 V for Li_2_S to nucleate and grow until the current dropped below 10^−5^ A. It took about 60,000 s and the energy was integrated to evaluate the capacities from deposition of lithium sulfide on various surfaces according to Faraday’s law.

The catalytic property of the BPQDs was further studied by CV test of symmetric cells. The CF/BPQD and CF symmetric cells were prepared by assembling identical electrodes with a Celgard 2400 membrane as separator, and 25 µL Li_2_S_4_ (0.2 mol L^−1^) tetraglyme electrolyte was added. CV was performance at CHI 660c electrochemical workstation at a scan rate of 1 mV s^−1^ between −1.0 and 1.0 V. LSV studies were conducted using CF/BPQD or CF as working electrode, lithium foil as counter electrode and 0.2 mol L^−1^ Li_2_S_4_ as catholyte. The cells were tested at electrochemical workstation between 1.7 and 2.8 V at a scan rate of 0.1 mV s^−1^.

### In situ characterization

In situ TEM experiment was conducted on a JEOL 2100-TEM equipped with a Nanofactory scanning tunneling microscope (STM) holder. A thin layer of Li metal was scratched on the tip of a sharp Cu rode as reference electrode, while PCNF/S/BPQD fibers were dispersed on the tip of another Cu wire as working electrode. During the transfer of the STM holder assembled with two electrodes in TEM chamber, Ar gas flow was used to protect Li metal from moisture and oxygen. A potential of −2.0 V vs. Li/Li_2_O was applied to working electrode to drive the lithiation reaction once a physical contact is confirmed between two electrodes. In situ TB experiment was conducted by sealing a PCNF/S/BPQD or PCNF/S cathode and a Li metal anode in a glass bottle filled with about 12.0 mL 1.0 mol L^−1^ LiTFSI 1,3-dioxolane: 1,2-dimethoxy (DOL: DME, 1/1 v/v) electrolyte. The transparent batteries were galvanostatically discharged at 0.10 mA for 8 h, when optical images were taken to show the color change of electrolyte. In situ EIS measurements were performed on a Bio-Logic VSP-300 analyzer. The cells were assembled using PCNF/S/BPQD or PCNF/S cathodes and Li metal anodes in an Ar-filled glove box, to be discussed below. After every 20 min galvanostatic discharge at 0.1 C, we held the cells for 15 min to reach equilibrium before performing the EIS measurement. The EIS was conducted at a perturbation amplitude of 5 mV in the frequency between 10 mHz and 100 kHz. The EIS spectra were simulated using Z-view software.

### Cell assembly and electrochemical performance

To measure of the electrochemical performance, the cathodes were prepared by mixing PCNF/S/BPQD composite, carbon black, and polyvinylidene fluoride binder at a mass ratio of 8:1:1 using NMP solvent. The slurry mixture was cast on aluminum foil and cut into discs (*ɸ* = 14 mm), giving rise to an average sulfur loading of about 2 mg cm^−2^. The cathodes were assembled into CR2032 coin cells using Li metal anode, 1 mol L^−1^ LiTFSI DOL/DME electrolyte with 1.0 wt% LiNO_3_ additive, and polyethylene membrane (Celgard 2400) separator in an Ar-filled glove box. The electrolyte added in the cell is 80 µL, and the electrolyte/sulfur ratios are about 26, 13, 8.7, and 6.5 mL g^−1^ for the electrodes with sulfur loadings of 2, 4, 6, and 8 mg cm^−2^, respectively. These values are comparable with previous reports^18,19,46^. The PCNF/BPQD/catholyte electrodes were prepared by dropping 45 µL 1.5 mol L^−1^ Li_2_S_6_ catholyte into PCNF/BPQD electrode substrate (1 × 1 cm^2^, about 3 mg cm^−2^ with 2 wt% of BPQD). The polysulfide catholyte was prepared by mixing stoichiometric amount of sulfur and Li_2_S in DOL/DME = 1/1 v/v with 1.85 mol L^−1^ LiTFSI and 0.2 mol L^−1^ LiNO_3_, according to the previous work^57^. The final electrode achieved a high sulfur loading of 13.2 mg cm^−2^ and a sulfur content of 81 wt%. Additional blank electrolyte was used to wet separator to achieve an E/S ratio of ~4 mL g^−1^. The cells were tested at different current densities between 1.7 and 2.8 V vs. Li^+^/Li on a LAND 2100CT battery tester. The post-mortem analysis was carried out by dissembling the cycled PCNF/S/BPQD electrodes in a glove box and washing with flooded amounts of DME to remove the salt and byproducts. Cycled electrodes were sealed in Ar-filled bottles before sending to SEM and TEM characterizations.

### Theoretical calculations

DFT calculations were performed with the Vienna Ab initio Simulation Package (VASP)^63,64^, within the generalized gradient approximation proposed by Perdew, Burke, and Ernzerhof (PBE)^65^. We used Grimme’s DFT-D2 method^66^ to correct for the vdW interaction poorly described by standard DFT. We studied the energetics of adsorbed LiPSs on two types of BP structures: 2D-free-standing monolayer and one-dimensional bilayers zig-zag terminated BP nanoribbons, as represented in Supplementary Fig. 16. It has been shown that ZZ terminations are lower in energy than their arm-chair counterpart^67^. Therefore, we assume that all the BPQD edges are ZZ-terminated. DFT simulations were performed with a kinetic energy cutoff of 500 eV and we used two *k*-points in the periodic directions to evaluate the integrals in the reciprocal space. Convergence was achieved when energy, force and stress reached a minimum of 5 × 10^−4^ eV, 5 × 10^−2^ eV Å^−1^, and 5 × 10^−2^ GPa, respectively. The potential energy surface corresponding to the adsorption of LiPS at the terrace or at the edge of BP is rough and presents multiple minima. Therefore, to find the lowest energy location of adsorbed LiPS on BP, we performed for each compound, ab initio molecular dynamics simulations at 300 K with a kinetic energy cutoff of 300 eV and a time step of 1.5 fs. Finally, we computed the binding energy *E*_b_ as:

1 $$E_{\mathrm{b}} = E_{{\mathrm{LiPS}}} + E_{{\mathrm{BP}}} - E_{{\mathrm{LiPS@BP}}}$$

with *E*_LiPS_, *E*_BP_, and *E*_LiPS@BP_ the energy of LiPSs, BP, and LiPSs adsorbed on the terrace site (or at the edge site) of BP, respectively. Following this definition, higher binding energy implies more favorable adsorption.

## Electronic supplementary material

Supplementary Information

Description of Additional Supplementary Files

Supplementary Movie 1

## Data Availability

The data that support the findings in this study are in the paper and/or the 524 Supplementary Information. Additional data are available from the authors upon reasonable 525 request.
