# Supplementary material for: Exceptional catalytic effects of black phosphorus quantum dots in shuttling-free lithium sulfur batteries
Source: Nat Commun. 2018 Oct 9;9:4164. doi: 10.1038/s41467-018-06629-9 (PMC6177446; doi:10.1038/s41467-018-06629-9)
Supplement: Supplementary file 1 — Supplementary Information [file 41467_2018_6629_MOESM1_ESM.pdf]

Supplementary Information for

Exceptional catalytic effects of black phosphorus quantum dots in shuttling-free lithium sulfur batteries

Xu *et al.*

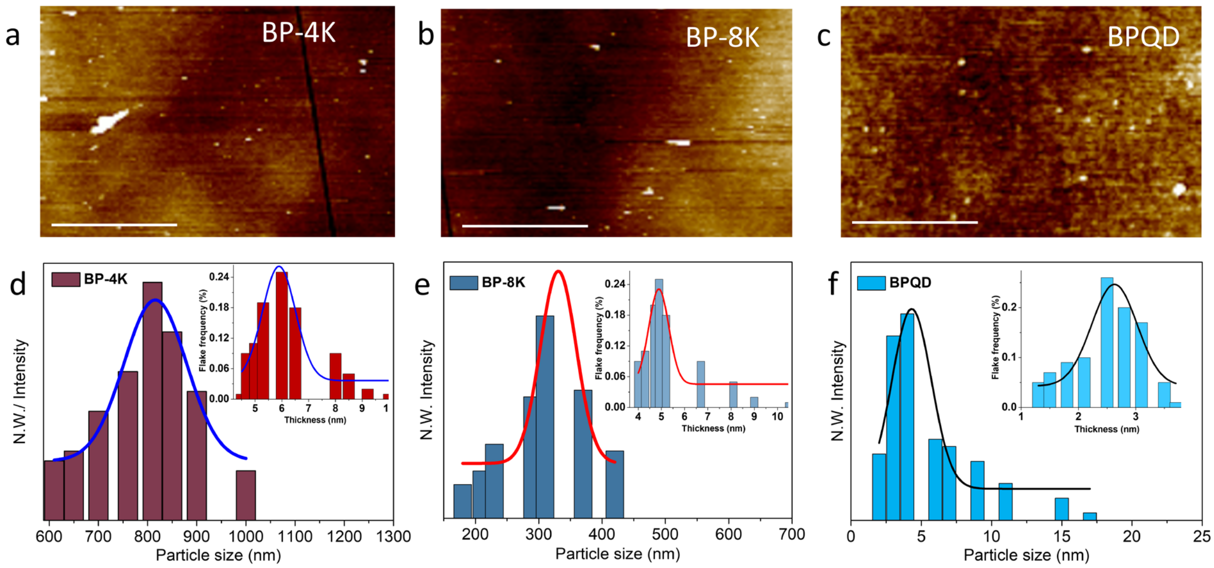

Supplementary Figure 1 | Dimensions of the BP flakes characterized by atomic force microscopy (AFM). Representative AFM images and particle size/thickness dimensions of (a, d) BP-4K, (b, e) BP-8K, and (c, f) BPQD. Inset of the (d-f) images are thickness distribution of corresponding BP flakes. Scale bars, 2 µm (a, b), 1 µm (c).

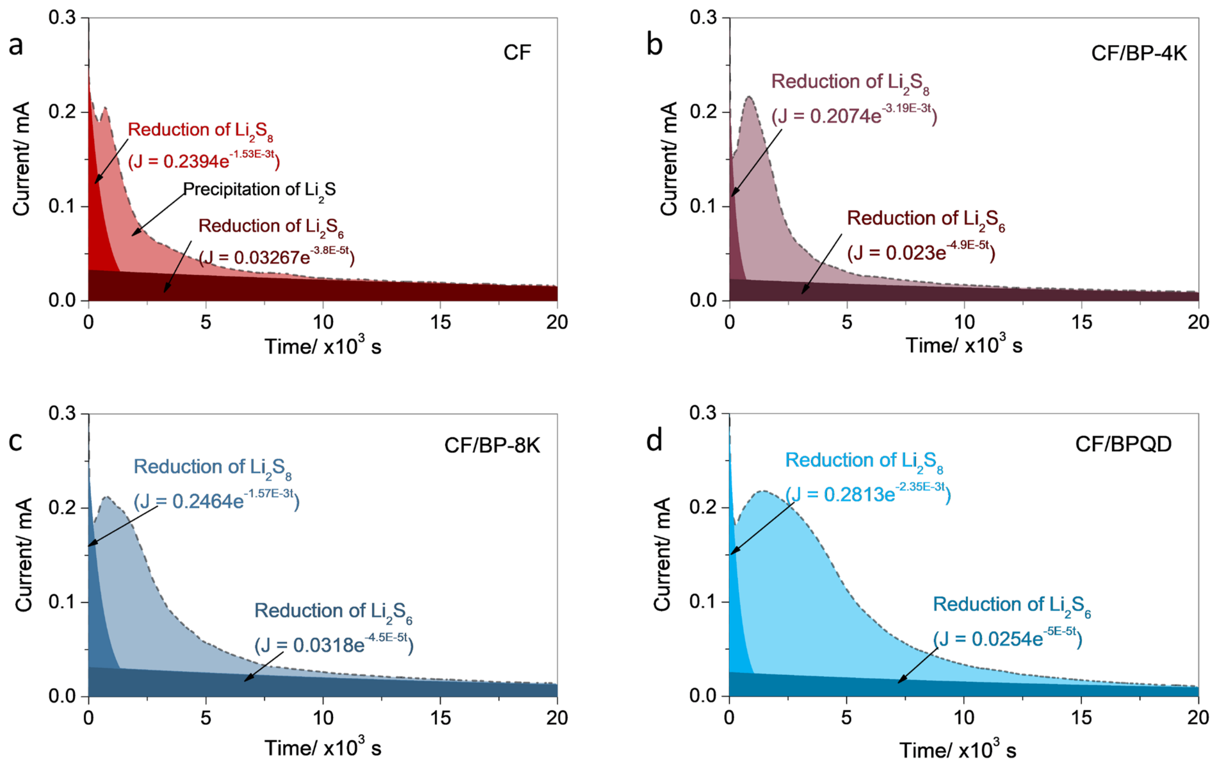

Supplementary Figure 2 | Fitting of current vs. time for potentiostatic discharge at 2.05 V on different surface. (a) carbon fiber (CF), (b) CF/BP-4K, (c) CF/BP-8K, and (d) CF/BPQD. The nucleation/growth rates of Li_2S on different substrates were fitted according to Faraday's low, similar with Zhang's work[1]. Specifically, the potentiostatic discharge curve at 2.06 V (the equilibrium potential) was fitted using the integration of the exponentially decayed curves, representing the reduction of Li_2S_8 and Li_2S_6, respectively. When an overpotential of 0.01 V was applied, Li_2S forms, giving rise to extra contribution to the overall current. The capacity from Li_2S formation was obtained by subtracting the capacity of Li_2S_8/Li_2S_6 reductions from overall capacities during the potentiostatic discharge curve.

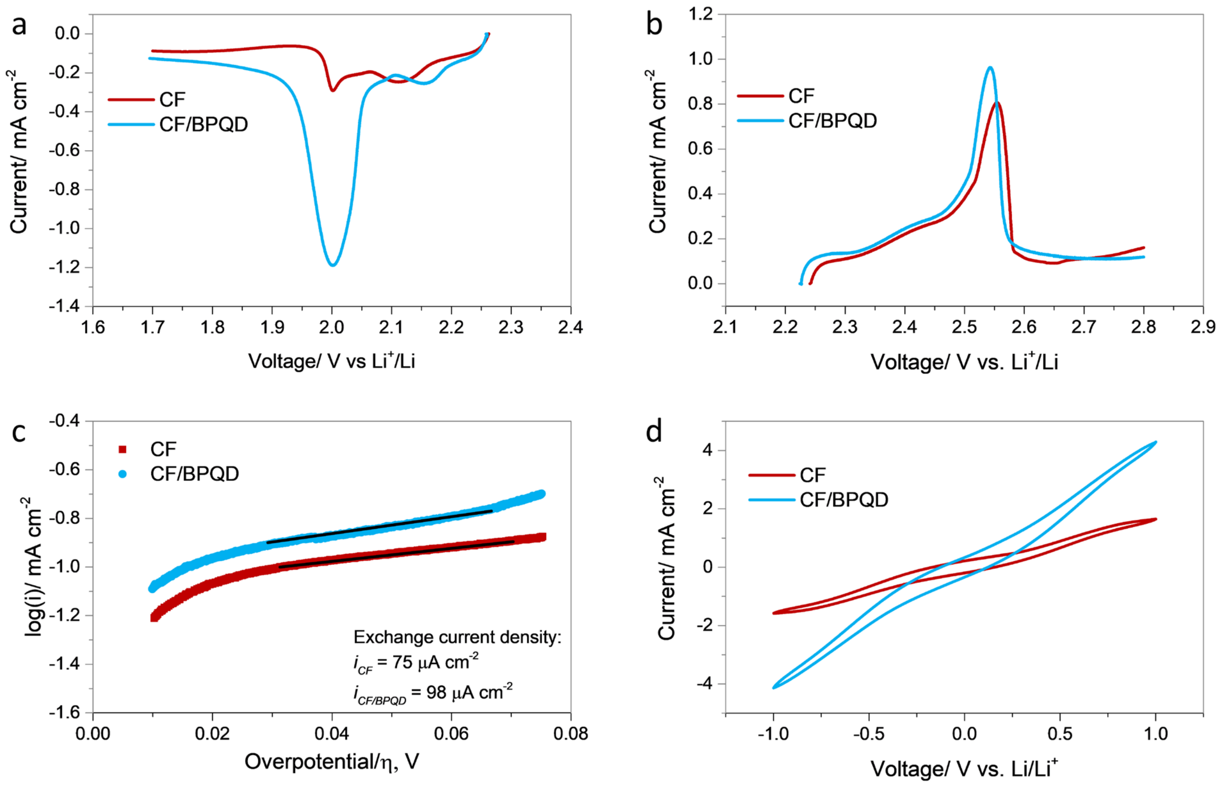

Supplementary Figure 3 | Linear sweep voltammogram (LSV) and cyclic voltammogram (CV) analyses of CF and CF/BPQD electrodes with Li_2S_4 catholyte. (a) Forward scan (from OCV to 1.7 V) and (b) backward scan (from OCV to 2.8 V) of CF and CF/BPQD electrodes with Li_2S_4 catholyte. (c) Tafel plots for CF and CF/BPQD electrodes in catholyte. The higher scanning current densities in (a) and (b) and the higher exchange current density in (c) of CF/BPQD than those for CF electrodes confirm the enhanced reaction kinetics for LiPS redox reactions by BPQD. (d) CV curves of Li_2S_4 symmetric cells using CF and CF/BPQD as current collectors. The higher redox current of CF/BPQD under a polarization of 1.0 V suggests enhanced redox of polysulfides by BPQDs.

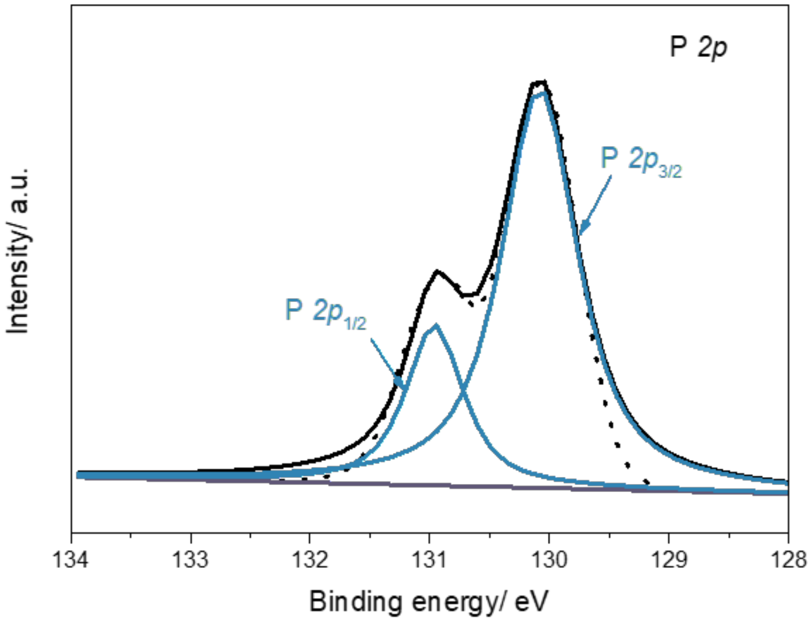

Supplementary Figure 4 | Deconvoluted XPS spectrum of the BPQDs. Fitted peaks at 130.9 eV and 130 eV are corresponding to P $2p_{1/2}$ and P $2p_{3/2}$, respectively.

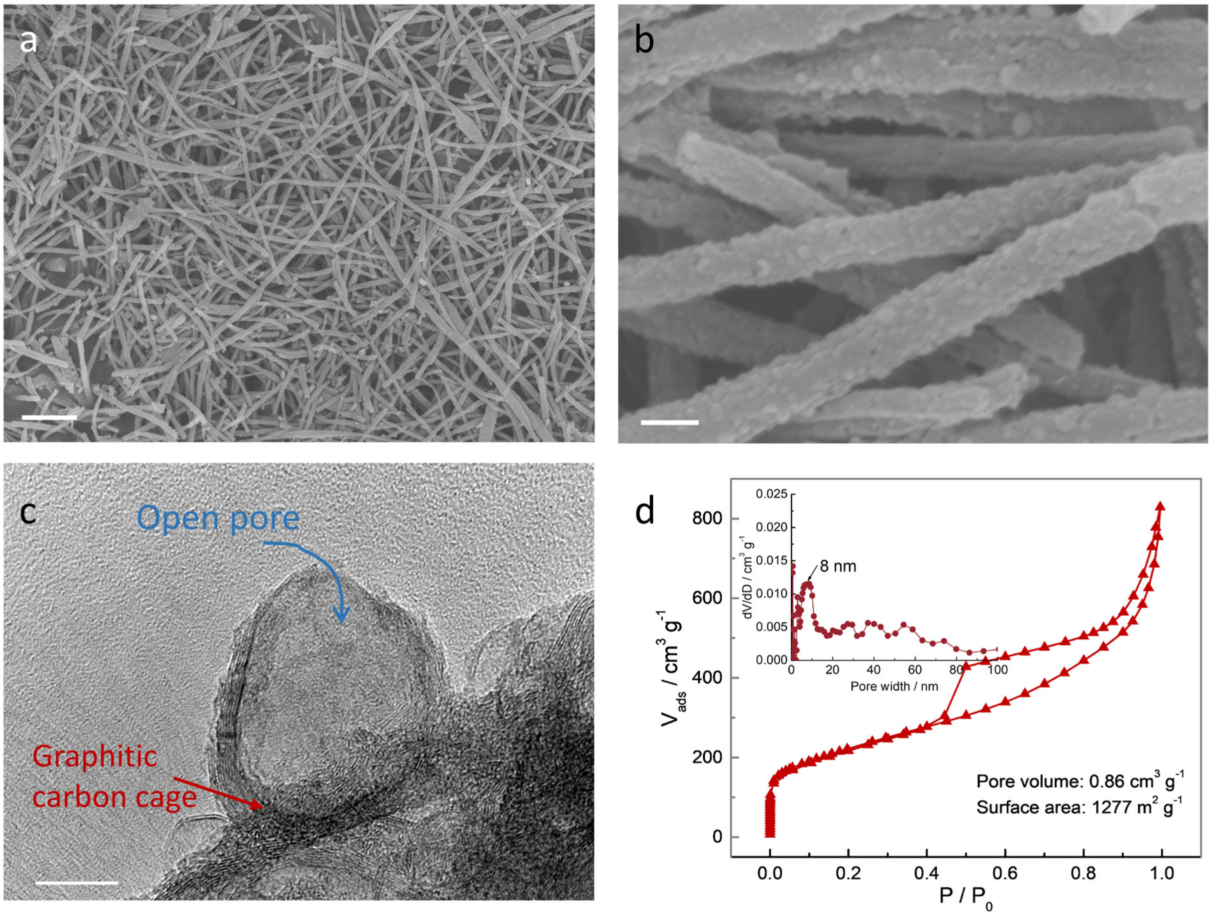

Supplementary Figure 5 | Characterizations of the electrospun porous carbon fiber (PCNF) host. (a and b) SEM images of the PCNF, showing rough surface and uniform diameter of the PCNFs. (c) HRTEM image of a hollow carbon sphere on PCNF, showing graphitic carbon layers and open pore. (d) BET isotherm and pore size distribution (inset of d) of the PCNF. The pore volume, surface area and average pore size on carbon walls are determined to be 0.86 cm^3 g^{-1}, 1277 m^2 g^{-1}, and 8 nm, respectively. Scale bars, 2 µm (a); 200 nm (b); 10 nm (c).

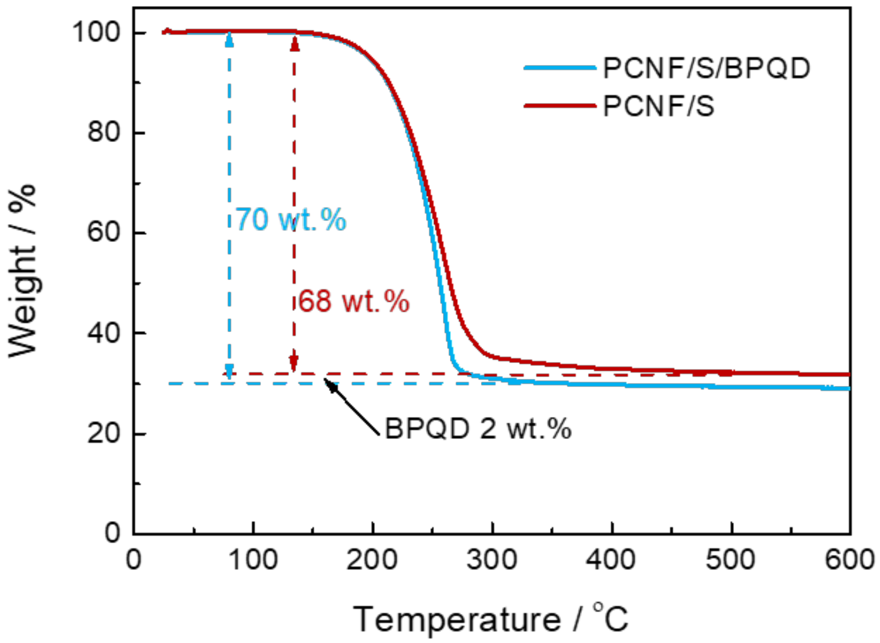

Supplementary Figure 6 | TGA curves of the PCNF/S and PCNF/S/BPQD composites. The PCNF/S/BPQD was prepared by mixing PCNF/S and BPQD in CS_2 solvent, thus the weight content of BPQD in PCNF/S/BPQD can be presented as the difference of sulfur content between PCNF/S and PCNF/S/BPQD. It is noted that large BP flakes sublimes at 300-400 °C in inert atmosphere[2] so that the degradation of BPQD may happen at below 300 °C.

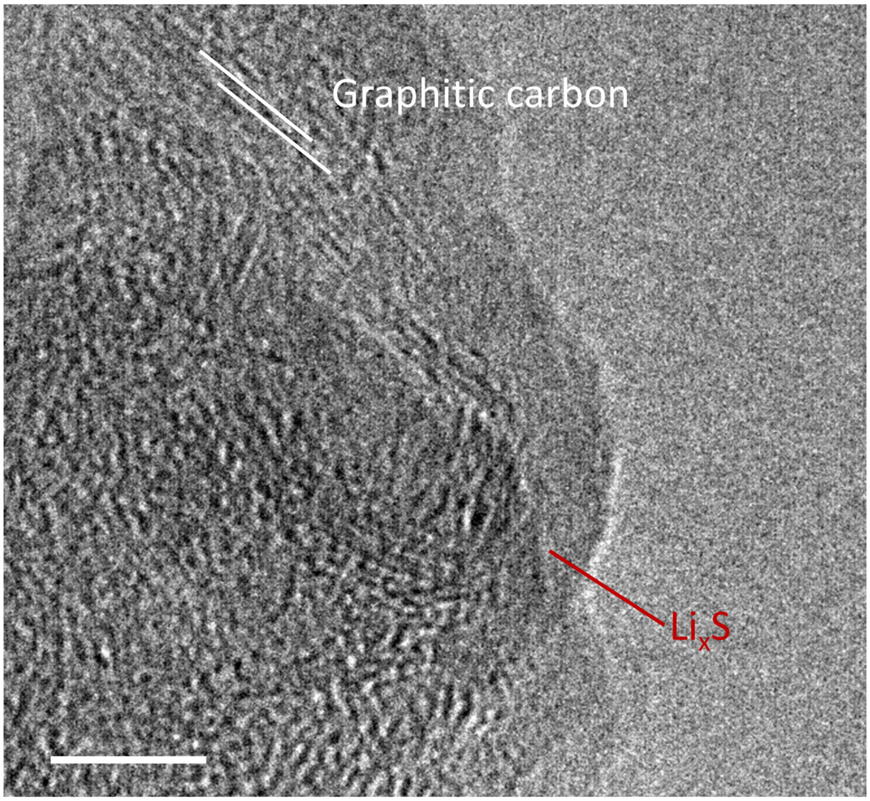

Supplementary Figure 7 | HRTEM image of the lithiated PCNF/S/BPQD. The lithiation product was squeezed out from graphitic carbon host due to the large volume expansion of sulfur during lithiation. Scale bar, 5 nm.

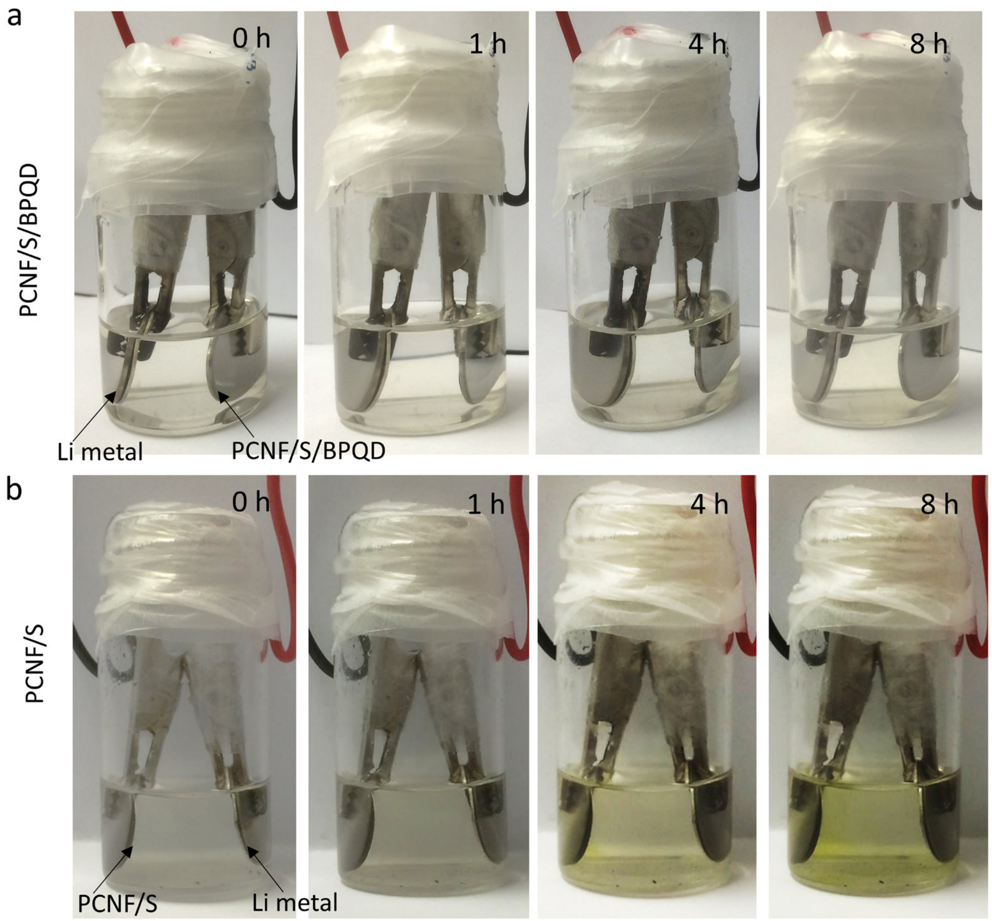

Supplementary Figure 8 | *In-situ* visual experiment displaying dissolution of LiPSs into the electrolyte after discharging (a) PCNF/S/BPQD and (b) PCNF/S cathodes.

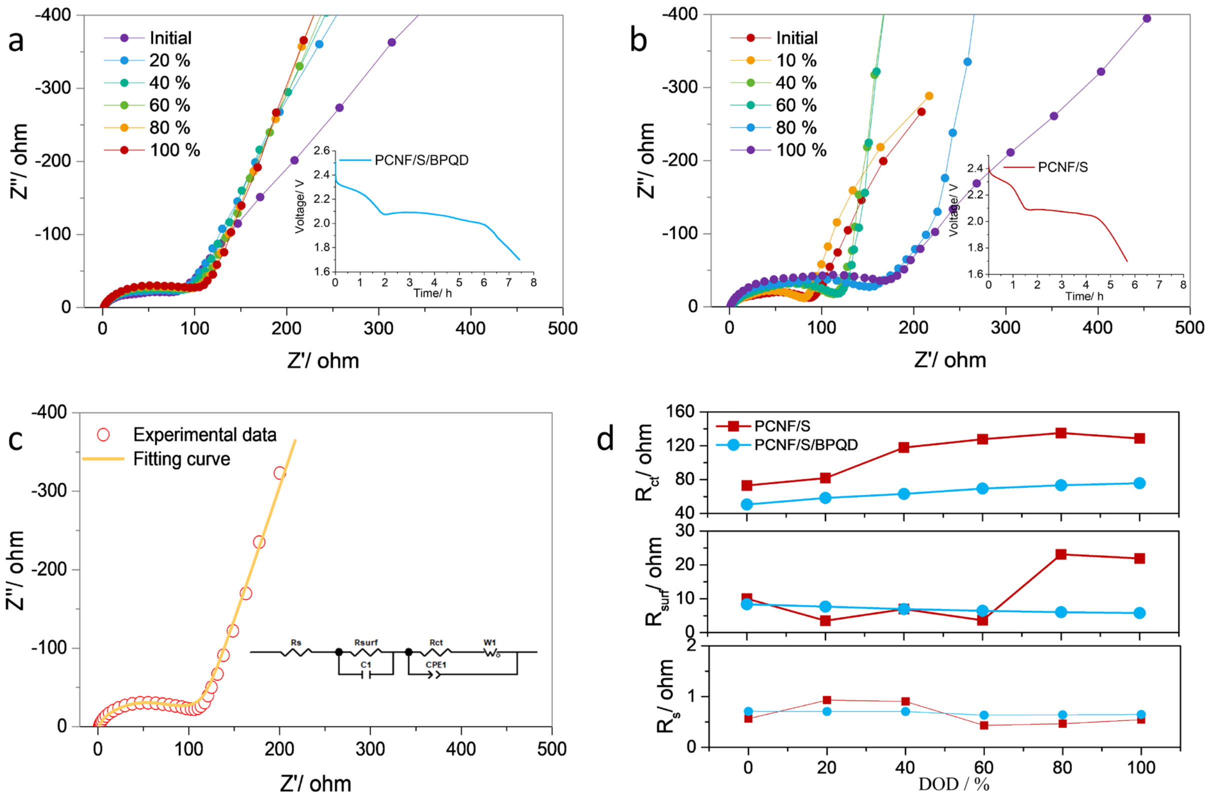

Supplementary Figure 9 | *In-situ* electrochemical impedance spectroscopy (EIS) characterization of the PCNF/S and PCNF/S/BPQD cathodes during initial lithiation. (a) Nyquist plots of the PCNF/S/BPQD electrode at different depth of discharge (DOD) (inset is the galvanostatic discharge curve for *in-situ* EIS test). (b) Nyquist plots of the PCNF/S electrode at different DOD and the discharge curve (inset). (c) Fitting result of the Nyquist plot of PCNF/S/BPQD electrode measured at the DOD of 100 %, inset is the equivalent circuit model. (d) Comparison of R_s, R_{surf} and R_{ct} obtained from (a) and (b) for the PCNF/S and PCNF/S/BPQD electrodes against DOD.

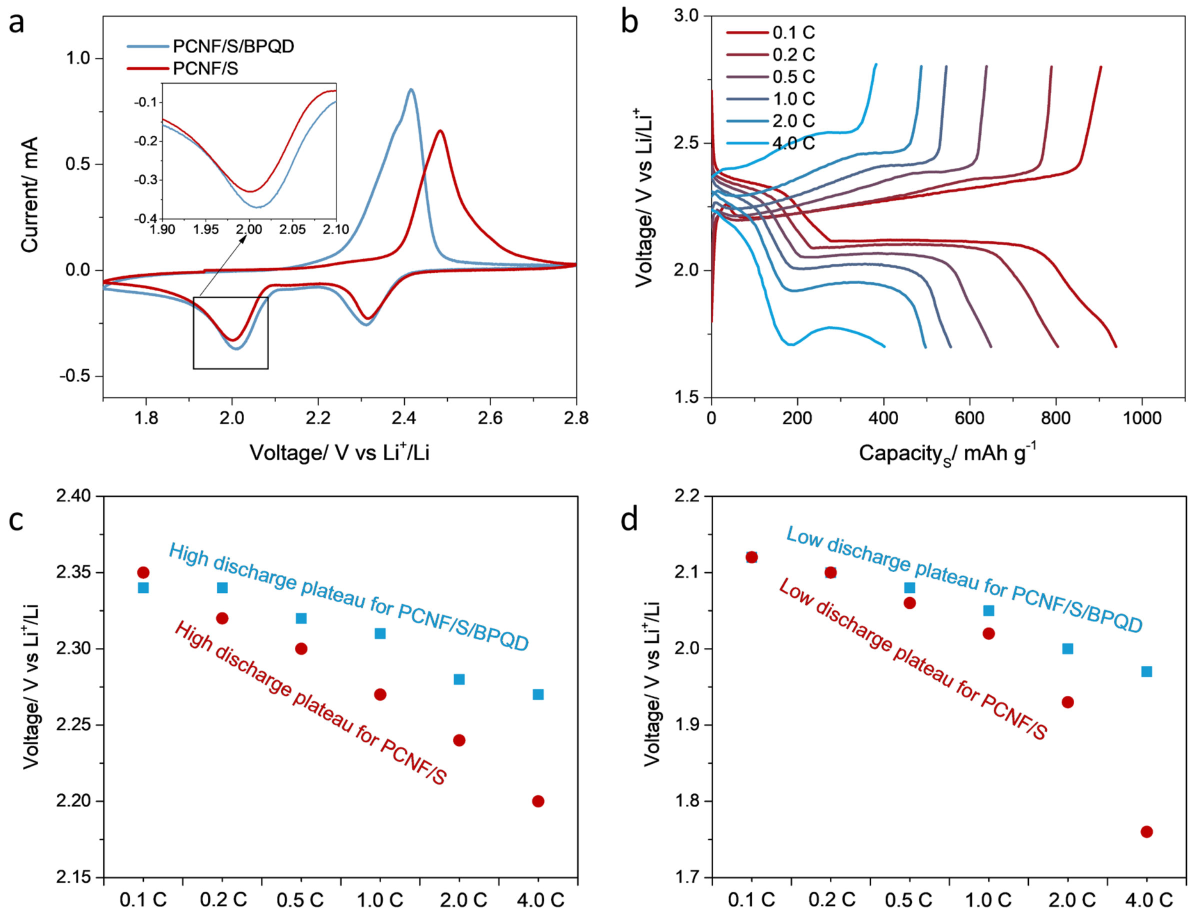

Supplementary Figure 10 | Electrochemical characteristics of the PCNF/S and PCNF/S/BPQD electrodes. (a) Initial CV curves of the PCNF/S and PCNF/S/BPQD electrodes at a scan rate of 1 mV s^{-1}. Inset is the magnification of the cathodic peak range 1.9-2.1 V. The PCNF/S/BPQD presents lower anodic peak at 2.4 V and higher cathodic peak at about 2.0 V than those for the PCNF/S, indicating lower polarization and faster conversion reaction kinetics for the PCNF/S/BPQD. (b) Discharge/charge curves of PCNF/S electrodes at different C rates. (c) High discharge plateau voltage and (d) low discharge plateau voltage of PCNF/S/BPQD and PCNF/S electrodes at different C rates.

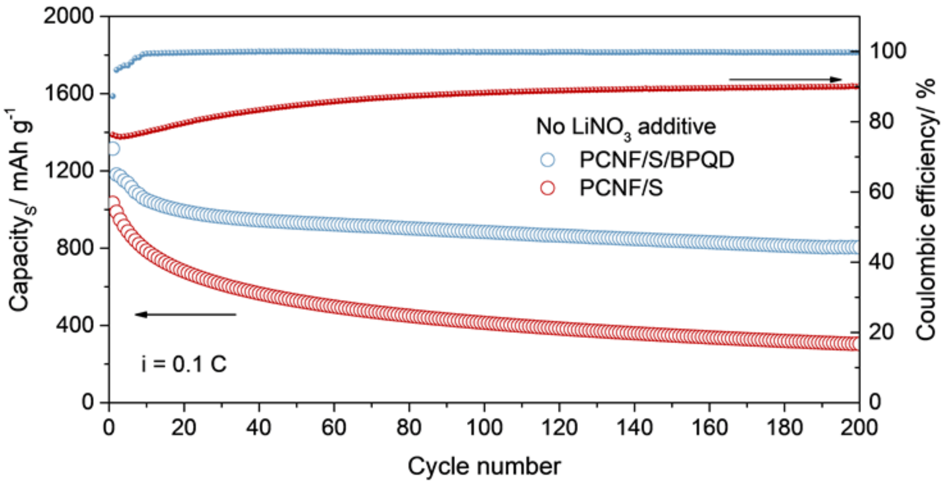

Supplementary Figure 11 | Cyclic performance of the PCNF/S and PCNF/S/BPQD electrodes in electrolyte without $LiNO_3$ additive. PCNF/S/BPQD presented a capacity retention of about 820 mAh g^{-1} after 200 cycles with Coulombic efficiencies of above 99 % after 10 cycles, which values are much higher than the 306 mAh g^{-1} and below 90 % for PCNF/S electrodes. This result indicates the effective immobilization of polysulfides by BPQD catalyst in $LiNO_3$-free electrolyte.

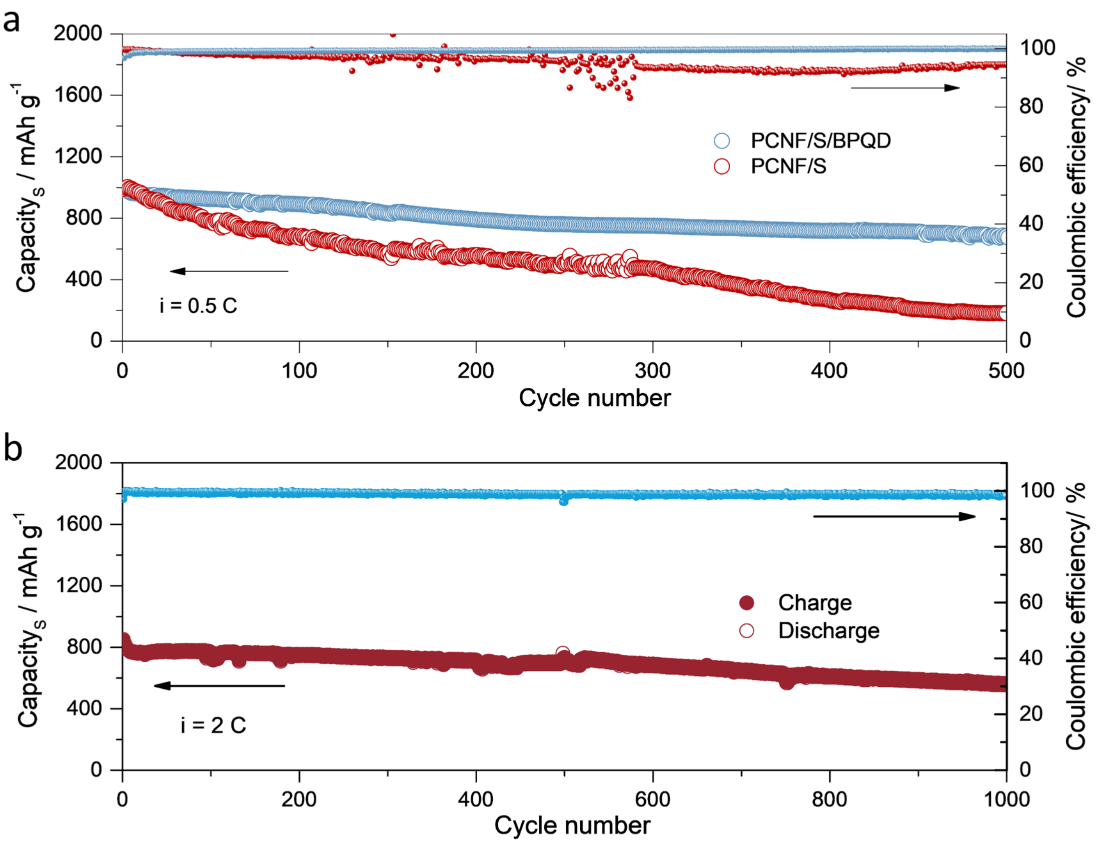

Supplementary Figure 12 | Long term cyclic performance of PCNF/S/BPQD electrodes. (a) Cyclic capacities and Coulombic efficiencies of PCNF/S and PCNF/S/BPQD at 0.5 C for 500 cycles, (b) long cycling for the PCNF/S/BPQD electrode at 2 C for 1000 cycles.

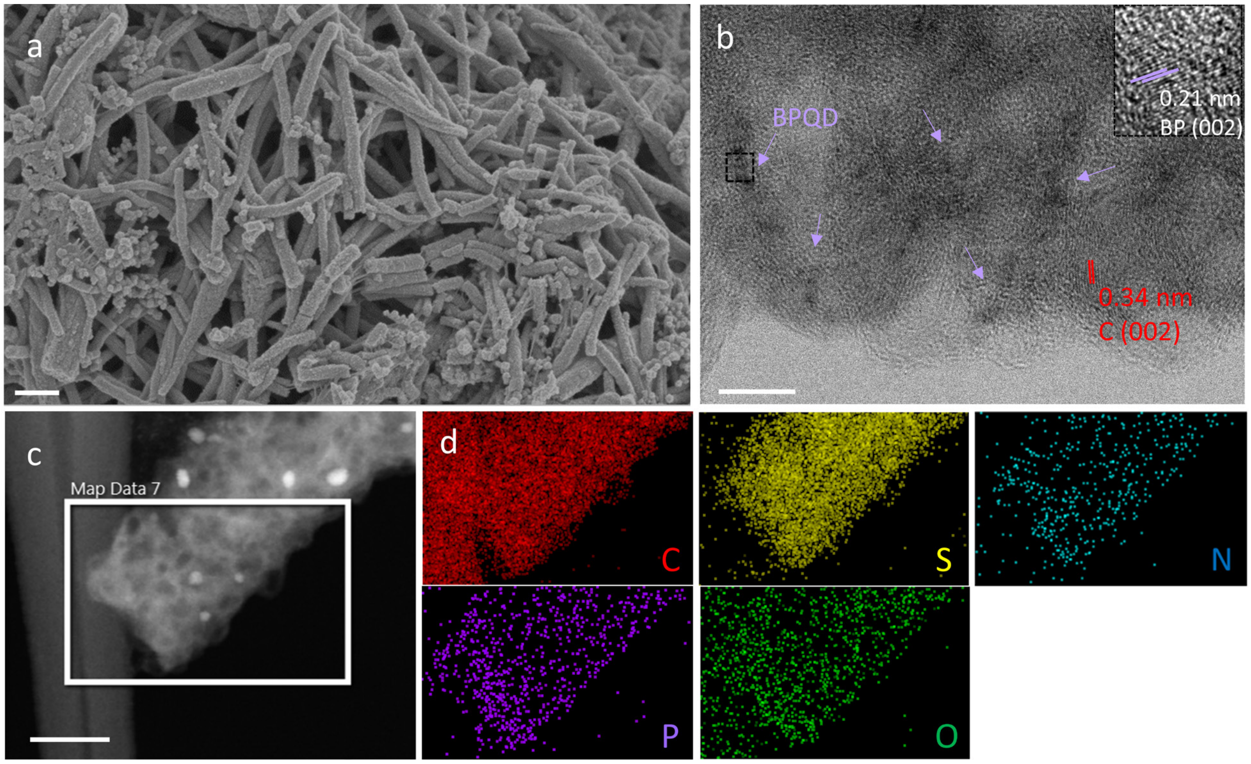

Supplementary Figure 13 | Analysis the post-cycled PCNF/S/BPQD electrodes in Figure 5b. (a) SEM image showing the intact fibrous morphology of PCNF/S/BPQD after 200 cycles at 0.1C, (b) HRTEM image presenting the uniformly dispersed BPQDs among graphitic carbon spheres, inset (b) showing the crystalline nature of BPQD, (c) STEM image and (d) C, S, P, O, and N elemental mappings showing uniform distribution of BPQD and the successful confinement of sulfur in PCNF host during cycling. Noted that the large white particles in (c) refer to few residual Fe_3C in PCNF after annealing and acid etching, consistent with our previous work [3]. Scale bars, 500 nm (a); 10 nm (b) and 50 nm (c).

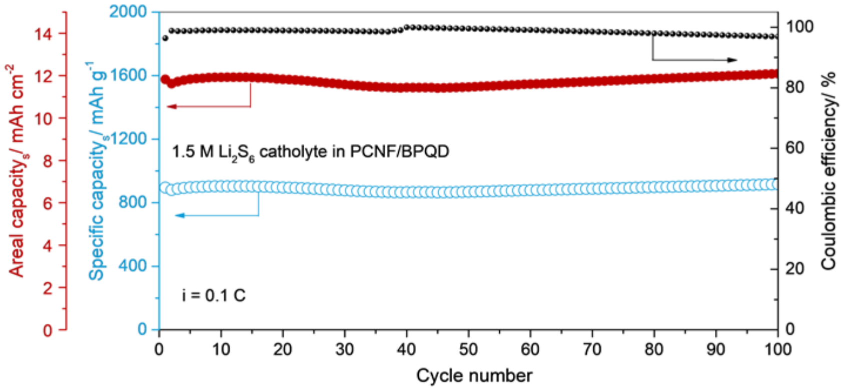

Supplementary Figure 14 | Cyclic performance of LSBs made from 1.5 M Li_2S_6 catholyte dropping on PCNF/BPQD. The catholyte-based electrodes possess a low E/S ratio of near 4 mL g^{-1} and a high sulfur loading of 13.2 mg cm^{-2}. The electrodes presented an areal capacity of about 12 mAh cm^{-2} at 0.1 C for 100 cycles.

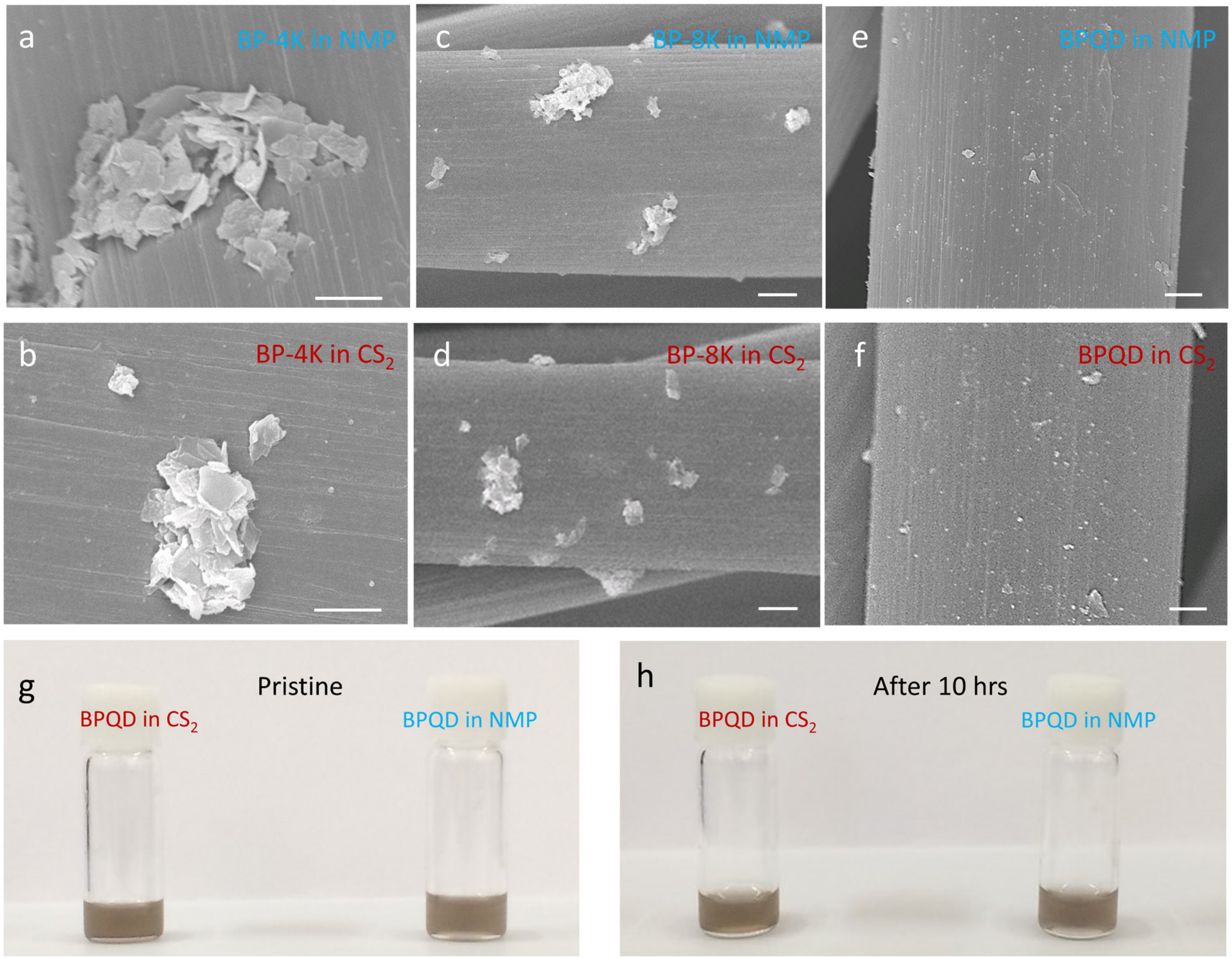

Supplementary Figure 15 | Comparison of the stability of BP nanostructures in CS_2 and NMP solvent. SEM images of (a, b) BP-4K, (c, d) BP-8K and (e, f) BPQD dispersed on carbon fiber substrates from NMP and CS_2 solutions; optical images of (g) pristine and (h) after 10 h of BPQDs CS_2 and NMP solutions. Comparison of the SEM and optical images reveals that CS_2 would not change the morphology and stability of BP nanostructures. The above results indicate that BPQDs are stable and ready to be composited with PCNF/S in CS_2 solvent. Scale bars, (a-f) 1 μm.

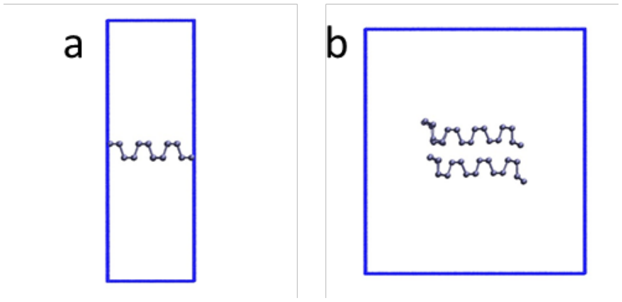

Supplementary Figure 16 | BP models used in this study. (a) 4×4 (48 atoms) free-standing monolayer and (b) (128 atoms) bilayer nanoribbons. We show in blue the contour of the simulation box used in the periodic calculations.

Supplementary Table 1 | Comparison of the BPQD with other catalyst materials for LSBs.

Material	Density/ g cm^{-3}	Conductivity/S cm^{-1}	Binding energy with Li_2S / eV
Pt[4]	21.4	9.4×10^4	/
Co[5]	8.9	1.7×10^5	/
TiN[6]	5.4	$1\text{-}10^4$	2.9
VN[7]	6.13	~70	3.7 (with Li_2S_6)
TiC[1]	4.93	/	2.75
WS_2[8]	7.5	(Semiconductor)	1.45
MoS_2[9]	5.06	(Semiconductor)	2.7 (for S), 0.87 for (Mo)
MnO_2[10]	5.03	(Insulator)	/
Fe_2O_3[11]	5.24	(Insulator)	4.8
TiN@TiO_2[12]	4.2-5.4	/	2.9-3.5 (with Li_2S_4)
Phosphorene[13]	2.69	4.5	2.5
BPQD (this work)	2.69	3-4.5[13,14]	2.5 (for terrace site)<br>4.2 (for edge site)

Supplementary Table 2 | Comparison of the electrochemical performance of Li-S batteries with different catalysts.

Catalyst	Cyclic performance	Rate performance	Areal capacity for high sulfur loading/ mAh cm^{-2}, E/S ratio	Reference
Pt/GO/Li_2S_6 catholyte	789 mAh/g after 100 cycles at 0.1 C	780 mAh/g at 0.2C	/	JACS 2015[4]
Co-N-GC/S	850 mAh/g after 200 cycles at 0.2 C	685 mAh/g at 2C, 565 mAh/g at 5C	/	EES 2016[5]
TiN/S	644 mAh/g after 500 cycles at 0.5 C	776 mAh/g at 1C	/	AM 2016 [6]
VN/GO/Li_2S_6 catholyte	917 mAh/g after 200 cycles at 1C	701 mAh/g at 3C	4.4 for 3 mg cm^{-2}, 3.76 after 100 cycles, ~31 mL g^{-1}	Nat Com. 2017 [7]
TiC/G/S	670 mAh/g after 100 cycles at 0.2 C	/	3.6 for 3.5 mg cm^{-2}, 2.3 after 100 cycles, ~22.6 mL g^{-1}	Angew2016 [1]
MoS_2/CNF/Li_2S_8 catholyte	800 mAh/g after 300 cycles at 0.5 C	~400 mAh/g at 2C	/	ACS Nano 2014 [9]
MnO_2/hollow CNF/S	~900 mAh/g after 100 cycles at 0.2C	690 mAh/g at 1C	3.2 for 3.5 mg cm^{-2}, 2.3 after 300 cycles	Angew 2015 [10]
TiN@TiO_2 interlayer	927 mAh/g after 300 cycles at 0.3C	682 mAh/g at 2C	~2.8 for 4.3 mg cm^{-2}, ~1.9 after 2000 cycles, 10.3 mL g^{-1}	EES 2017[12]
Phosphorene/CNF/Li_2S_6 catholyte	660 mAh/g after 500 cycles at 1C	785 mAh/g at 3C	~5 for ~5 mg cm^{-2}, ~10 mL g^{-1}	AM 2017[13]
WS_2/Li_2S_6 catholyte	596 mAh/g after 350 cycles at 0.5 C	380 mAh/g at 1C	/	JACS 2017[15]
TiS_2/S/G/CNT	546 mAh/g after 300 cycles at 0.5 C	~420 mAh/g at 4C	/	PANS 2017[16]
CoS_2/S/G/CNT	581 mAh/g after 300 cycles at 0.5 C	~500 mAh/g at 4C	/	
VS_2/S/G/CNT	701 mAh/g after 300 cycles at 0.5 C	~590 mAh/g at 4C	/	
Phosphorene separator	~800 mAh/g after 100 cycles at ~0.24C	623 mAh/g at ~2C	/	AM 2016[14]
PCNF/S/BPQD	1073 mAh/g after 200 cycles at 0.1C 589 mAh/g after 1000 cycles at 2C	784 mAh/g at 4C	4.9 for 8 mg cm^{-2}, 4.4 after 200 cycles, ~6.5 mL g^{-1}	This Work

Supplementary Note 1 | Yield analysis of BPQD synthesis

We evaluated the yield of BPQD produced by sonication-centrifugation method in this work. The BPQDs were obtained by probe-sonicating BP sheets. The BP sheets were prepared by grinding and sonicating bulk BP crystals in NMP solvent. Typically, 0.1 g bulk BP was added in NMP solvent and grinded in a mortar for 20 min. The mixture was then transferred to a sonicator (Sonics Vibra-cell VC 750 tip sonicator) for ultra-sonication for 8 h in an ice-bath. The as-obtained solution was centrifuged at a rate of 3000 rpm for 30 min to remove the un-exfoliated BP crystals at bottom and obtain the BP sheet in suspension. The mass of the as-obtained BP sheet powder is 10-15 mg, rendering a yield of 10-15 %, similar with the values in literature[17,18]. Then, the BP sheet suspension was further sonicated to fully downsize the BP sheets to QDs. The morphological evolution of BP sheets after different sonication time are shown in Fig. 17 (Supplementary Information), which indicates that BP sheets can be fully transferred to QDs after 2 h's sonication. We also measured the weight of BPQD powder after centrifugation and drying, which is about 98% of the BP sheets we used. Thus, we can conclude that the yield of BPQD in this work is 9.8- 14.7%, depending on the yield of BP sheets. We believe that the yield of BPQD can be further improved when the yield of BP sheets is increased in the future.

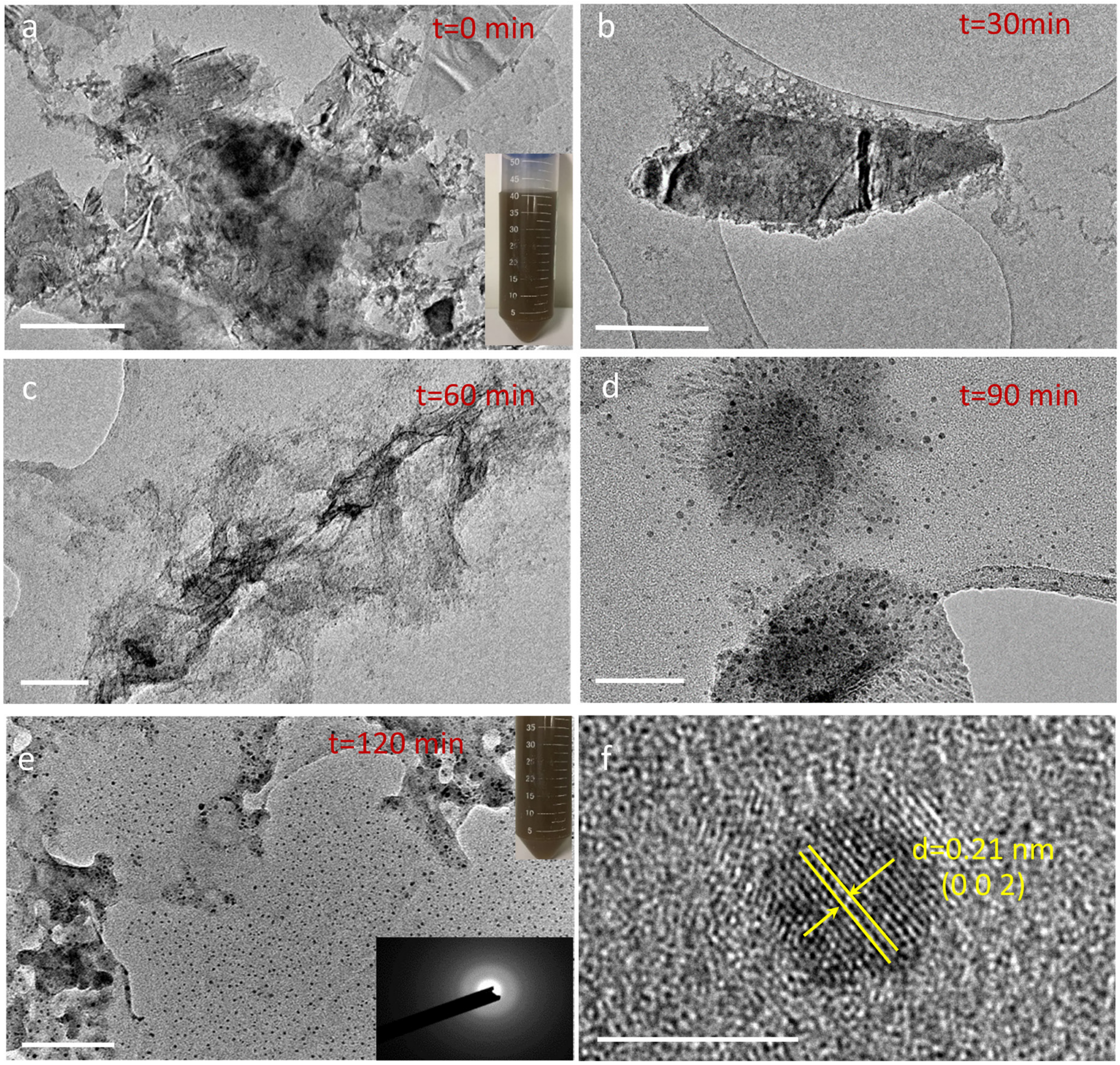

Supplementary Figure 17 | Procedures of preparing BPQD from BP sheets. TEM images of BP sheets after sonicating for (a) 0 min, (b) 30 min, (c) 60 min, (d) 90 min and (e) 120 min, (f) HRTEM image of an individual BPQD with a particle size of about 3 nm and good crystallinity. Inset of (a) and (e) show the optimal images of BP sheets and QDs in NMP solution. The selected area electron diffraction (SEAD) pattern of crystalline BPQDs is presented inset of (e). Scale bars, 0.5 μm (a, b); 200 nm (c); 50 nm (d); 100 nm (e) and 5 nm (f).

Supplementary References:

1. Peng, H. J. *et al.* Enhanced electrochemical kinetics on conductive polar mediators for lithium–sulfur batteries. *Angew. Chem. Int. Ed.* **55,** 12990–12995 (2016).
2. Liu, X., Wood, J. D., Chen, K. S., Cho, E. & Hersam, M. C. In situ thermal decomposition of exfoliated two-dimensional black phosphorus. *J. Phys. Chem. Lett.* **6,** 773–778 (2015).
3. Xu, Z.-L. *et al.* In situ TEM study of volume expansion in porous carbon nanofiber/sulfur cathodes with exceptional high-rate performance. *Adv. Energy Mater.* **7,** 1602078 (2017).
4. Al Salem, H., Babu, G., V. Rao, C. & Arava, L. M. R. Electrocatalytic polysulfide traps for controlling redox shuttle process of Li-S batteries. *J. Am. Chem. Soc.* **137,** 11542–11545 (2015).
5. Li, Y.-J., Fan, J.-M., Zheng, M.-S. & Dong, Q.-F. A novel synergistic composite with multi-functional effects for high-performance Li–S batteries. *Energy Environ. Sci.* **9,** 1998–2004 (2016).
6. Cui, Z., Zu, C., Zhou, W., Manthiram, A. & Goodenough, J. B. Mesoporous titanium nitride-enabled highly stable lithium-sulfur batteries. *Adv. Mater.* **28,** 6926–6931 (2016).
7. Sun, Z. *et al.* Conductive porous vanadium nitride/graphene composite as chemical anchor of polysulfides for lithium-sulfur batteries. *Nat. Commun.* **8,** 14627 (2017).
8. Lei, T. *et al.* Multi-functional layered WS_2 nanosheets for enhancing the performance of lithium-sulfur batteries. *Adv. Energy Mater.* **7,** 1601843 (2017).
9. Lin, H. *et al.* Electrocatalysis of polysulfide conversion by sulfur-deficient MoS_2 nanoflakes for lithium–sulfur batteries. *Energy Environ. Sci.* **10,** 1476–1486 (2017).
10. Li, Z., Zhang, J. & Lou, X. W. Hollow carbon nanofibers filled with MnO_2 nanosheets as efficient sulfur hosts for lithium-sulfur batteries. *Angew. Chem. Int. Ed.* **54,** 12886–12890

(2015).

11. Zheng, C. *et al.* Propelling polysulfides transformation for high-rate and long-life lithium–sulfur batteries. *Nano Energy* **33,** 306–312 (2017).

12. Zhou, T. *et al.* Twinborn TiO_2 –TiN heterostructures enabling smooth trapping–diffusion–conversion of polysulfides towards ultralong life lithium–sulfur batteries. *Energy Environ. Sci.* **10,** 1694–1703 (2017).

13. Li, L. *et al.* Phosphorene as a polysulfide immobilizer and catalyst in high-performance lithium-sulfur batteries. *Adv. Mater.* **29,** 1602734 (2017).

14. Sun, J. *et al.* Entrapment of polysulfides by a black-phosphorus-modified separator for lithium–sulfur batteries. *Adv. Mater.* **28,** 9797–9803 (2016).

15. Babu, G., Masurkar, N., Al Salem, H. & Arava, L. M. R. Transition metal dichalcogenide atomic layers for lithium polysulfides electrocatalysis. *J. Am. Chem. Soc.* **139,** 171–178 (2017).

16. Zhou, G. *et al.* Catalytic oxidation of Li_2S on the surface of metal sulfides for Li−S batteries. *Proc. Natl. Acad. Sci. USA* **114,** 840–845 (2017).

17. Zhao, W. *et al.* Large-scale, highly efficient, and green liquid-exfoliation of black phosphorus in ionic liquids. *ACS Appl. Mater. Interfaces* **7,** 27608–27612 (2015).

18. Chen, L. *et al.* Scalable clean exfoliation of high-quality few-layer black phosphorus for a flexible lithium ion battery. *Adv. Mater.* **28,** 510–517 (2016).
