# Supplementary material for: Exceptional catalytic effects of black phosphorus quantum dots in shuttling-free lithium sulfur batteries
Source: Nat Commun. 2018 Oct 9;9:4164. doi: 10.1038/s41467-018-06629-9 (PMC6177446; doi:10.1038/s41467-018-06629-9)
Supplement: Supplementary file 2 — Description of Additional Supplementary Files [file 41467_2018_6629_MOESM2_ESM.pdf]

Description of Additional Supplementary File

File name: Supplementary Movie 1

Description: *In-situ* TEM observation of the initial lithiation of the PCNF/S/BPQD fiber with the operating bias of 2 V. The video is played 12 times of real speed.
